# Ramping up the heat: Induction of systemic and pulmonary immune responses and metabolic adaptations in mice

**DOI:** 10.14814/phy2.71164

**Published:** 2026-11-02

**Authors:** Laura E. Dean, Andrea Adamcakova‐Dodd, Hans‐Joachim Lehmler

**Affiliations:** ^1^ Department of Occupational and Environmental Health, College of Public Health University of Iowa Iowa City Iowa USA

**Keywords:** gradual heat exposure, immunometabolism, metabolomics, pulmonary immune responses, sex differences, transcriptomics

## Abstract

Heatwaves are an increasing public health concern, yet most animal studies use sudden extreme heat exposure that poorly reflects real‐world conditions. This study examined the effects of gradual exposure to extreme heat on mice. Adult male and female C57Bl/6 mice were randomly assigned to heat‐exposed, control, or pair‐fed experimental groups. Heat‐exposed mice underwent a single 8‐h temperature ramp from 20°C to 38°C; control and pair‐fed mice remained at ambient temperature. Multi‐omics analyses after heat exposure included cytokine profiling in serum and lung, cecal microbiome characterization, lung transcriptomics, and serum metabolomics. Heat exposure altered several serum and lung cytokines (IL‐17α, MIP‐1α, MIP‐1β, IL‐1α, IL‐12(p40), and RANTES). Random forest analysis identified microbial taxa that distinguished experimental groups, with a reduction in Lactobacillus observed in males; however, these effects were not statistically significant. Lung transcriptomics revealed changes in immune‐related gene expression, particularly in pathways associated with B‐cell activation. Serum metabolomics indicated modest disruptions in amino acid and energy metabolism, with enrichment of the glycine, serine, and threonine metabolism pathway. Integrated analyses identified associations among cytokines, gene expression, and bile acid‐related metabolites. These findings demonstrate that gradual exposure to extreme heat triggers systemic and pulmonary responses in mice.

## Graphical abstract

Gradual heat exposure (20°C–38°C) triggered immune, lung, and metabolic changes in mice, revealing systemic responses that may better model the biological effects of real‐world heatwaves.

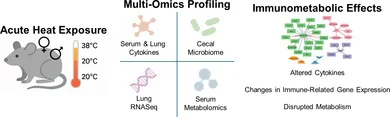

## 1 INTRODUCTION

Extreme heat events pose a significant health risk to humans, livestock, and wildlife (Dou et al., 2021; Ebi et al., 2021; Meador et al., 2020; Xiao et al., 2020). Extreme heat is linked to a range of heat‐related illnesses, including cardiovascular disorders (Daanen & Herweijer, 2015; Ebi et al., 2021; Folkerts et al., 2020; Périard et al., 2016). Exposure to extreme heat can trigger systemic inflammation that affects both the brain and peripheral organs, ultimately leading to systemic inflammatory response syndrome (Leon & Helwig, 2010). Heatwaves also increase hospital visits for respiratory illness (Chai et al., 2020; Ma et al., 2025), affect morbidity and mortality among patients with chronic lung diseases (Konstantinoudis et al., 2022; Scheerens et al., 2022; Silveira et al., 2023), and exacerbate the adverse effects of air pollution (Grigorieva & Lukyanets, 2021; Ruiz‐Páez et al., 2025). Therefore, major health organizations have prioritized further research into the health impacts of extreme heat to develop effective adaptation and mitigation strategies (Crowley et al., 2016; Huang et al., 2015; McCormack et al., 2016; Patz et al., 2014).

Thermoregulatory strategies differ markedly across mammalian species (Gordon, 2012). For example, in humans and some other large animals, thermoregulation during acute heat exposure can involve various physiological responses, such as vasodilation, increased heart rate to facilitate heat transfer to the skin, and enhanced sweat production for evaporative cooling (Ebi et al., 2021; Kang et al., 2023; Lepeule et al., 2018; Navas‐Martín et al., 2024). Mice, which are poor evaporative coolers and do not rely on whole‐body sweating, respond to heat by increasing heat dissipation, reducing metabolic heat production, and rising core temperature (Gordon, 2012; Škop et al., 2020). Because of these species differences, findings in mice can inform but cannot directly equate to the human physiological response to heat. Acute heat can also compromise respiratory function, disrupt endothelial barriers, and trigger systemic inflammation, leading to tissue damage and heat stress (Ebi et al., 2021; Miao et al., 2022). The lung, a primary interface between the external environment and internal physiology (Mori & Cardoso, 2014), is key to understanding how heat exposure can contribute to broader pathophysiological outcomes, including heat‐induced inflammatory changes (Liu et al., 2025). During heatwaves, activation of the heat shock response and subsequent increase in heat shock protein expression can improve cellular protection against heat stress (Sartori & Scherrer, 2003; Wheeler & Wong, 2007). In humans, this cellular‐level adaptation complements systemic physiological changes, such as improved sweating and cardiovascular responses, enhancing overall heat tolerance (Racinais et al., 2019); the corresponding mechanisms in mice, which lack whole‐body sweating, are less well characterized.

The effects of sudden, extreme heat exposure have been investigated extensively in animal models, such as rats (Chauhan et al., 2017; Dou et al., 2021), mice (Lee et al., 2015; Leon et al., 2005; Miyamoto et al., 2021; Roths et al., 2024), or livestock (Kang et al., 2023; Lebret et al., 2023; Mayorga et al., 2020; Rajamanickam et al., 2025). Similarly, laboratory studies of human responses to extreme heat use sudden heat exposure paradigms (Meade et al., 2024). These paradigms mimic abrupt transitions, like moving from air conditioning to outdoor heat. However, few studies address gradual temperature increases that better reflect the real‐world environmental heat‐exposure profiles (Ahrens, 2009) encountered by, for example, farmers (Elliott et al., 2022), athletes (Sawka et al., 1993), and military personnel (Alele et al., 2020; Parsons et al., 2019) during gradual heat exposures in outdoor settings. Importantly, populations vulnerable to extreme heat, such as children and elderly individuals (Basu, 2009; D'Amato & D'Amato, 2023; Leon & Helwig, 2010; Lepeule et al., 2018), face elevated risks when residing in buildings without adequate air conditioning (Baniassadi et al., 2019) because indoor temperatures during extreme heat events correlate with outdoor temperatures (Nguyen et al., 2014).

In this study, we use a systems biology approach to investigate the pulmonary and systemic effects of a gradual ambient temperature ramp from 20°C to 38°C in mice, designed to approximate the gradual temperature rise of an environmentally extreme heat event. We comprehensively assess serum and lung inflammatory markers, cecal microbiota, lung gene expression, and serum metabolite profiles to elucidate how this ambient temperature increase influences these molecular parameters. Our findings suggest that this gradual heat exposure, hereafter only referred to as heat exposure, is associated with systemic and pulmonary immune and metabolic responses in male and female mice.

## 2 MATERIALS AND METHODS

### 2.1 Animals and exposure

Animals were housed in an Association for Assessment and Accreditation of Laboratory Animal Care International (AAALAC)‐accredited facility at the University of Iowa under controlled conditions (23°C, 55% relative humidity, 12 h light/dark cycle) for 3 weeks prior to heat exposure. Bedding was mixed to synchronize the microbiome before exposure twice a week throughout the acclimation period (Miyoshi et al., 2018; Van Loo et al., 2000). Adult, 10‐week‐old C57Bl/6 mice (*n* = 24 females, 20 males) were obtained from Jackson Labs (Bar Harbor, ME, USA). Males (25.5 ± 1.3 g) and females (20.4 ± 1.3 g) were randomly assigned to one of three experimental groups (heat‐exposed (*n* = 8 females and 7 males), control (*n* = 8 females and 7 males), or pair‐fed (*n* = 8 females and 6 males)) using the random number generator function of Microsoft Excel (version 2308). Females were estrus synchronized using 5 IU of pregnant mare serum (Prospec, East Brunswick, NJ, USA) and human chorionic gonadotropin (Sigma Aldrich, St. Louis, MO, USA) 5 days and 3 days, respectively, before heat exposure to eliminate estrous cycling as a confounding factor (Ajayi & Akhigbe, 2020; Goldman et al., 2007).

Animals were singly housed and had access to Teklad‐7913 chow (ENVIGO, Indianapolis, IN, USA) and water ad libitum, except pair‐fed mice, which received access to the same amount of food and water that heat‐exposed mice consumed during the 8‐h heat event, to determine whether observed effects are due to heat exposure or variations in diet and hydration. Cages were weighed before heat exposure when empty, with water added, and with food added. After exposure, they were weighed in reverse order to determine the amount of food and water consumed. Measurement issues associated with feed and water intake resulted in the exclusion of one female control mouse and two female pair‐fed mice from the feed consumption analysis, and one male heat‐exposed mouse and two female pair‐fed mice from the water consumption analysis.

Heat‐exposed mice were placed in an incubator (Powers Scientific, Inc., Doylestown, PA, USA), and the temperature was gradually increased from 20°C to 38°C over 8 h during a single session (ZT3 to ZT11; 9:00–17:00 CST). This temperature ramp approximates a diurnal temperature rise at the start of a heatwave, progressing from a morning minimum to a mid‐to‐late‐afternoon maximum (Ahrens, 2009); the temperature maximum of 38°C falls within the range of maximum ambient temperatures observed during extreme heat events (Barriopedro et al., 2011; Kendon et al., 2020; Nitschke et al., 2011). We selected the 38°C maximum based on animal care concerns. Control and pair‐fed mice were placed on a benchtop in the same room as the incubator and maintained at ambient temperature (23°C) during the 8‐h exposure. The relative humidity in the incubator was 61% ± 8%, and in the animal room, it was 55% ± 1% throughout the exposures. Core body temperature, physiological parameters, and behavioral changes were not recorded during heat exposure to avoid sudden drops in temperature when opening the incubator doors. Mice were euthanized via cervical dislocation and decapitation immediately after the 8‐h exposure. Trunk blood was collected for serum (stored at −80°C) for further analyses. Lungs were flash frozen (stored at −80°C) for cytokine analysis or placed in RNAlater and stored at −20°C. Cecum contents were flash‐frozen for microbiome analysis and stored at −80°C. All data reported in this manuscript are publicly available on Iowa Research Online (Dean et al., 2026).

### 2.2 Cytokine analysis

Lung tissues were homogenized in T‐PER buffer (Thermo Scientific, Waltham, MA, USA) at a ratio of 1 mg of tissue to 20 μL of buffer for cytokine and chemokine measurements. Homogenates were centrifuged at 10,000 × *g* for 5 min at 4°C, and the resulting supernatant was collected. Total protein concentrations were determined using the Pierce BCA Protein Assay Kit (catalog #23225, Thermo Scientific) following the manufacturer's instructions.

Cytokine and chemokine levels (Table S1) were quantified in both serum and lung homogenates using the Bio‐Plex Pro Mouse Cytokine 23‐plex assay (Bio‐Rad Laboratories; cat. no. M60009RDPD) according to the manufacturer's protocol. A Luminex 200 system (Bio‐Rad Laboratories) was employed for data acquisition and analysis (Areecheewakul et al., 2023). Serial dilutions of the lyophilized cytokine standard were prepared using the provided assay diluent. For lung homogenates, a 1:1 mixture of assay diluent and T‐PER reagent was used. Serum samples heat‐exposed (*n* = 8 females and 6 males), control (*n* = 7 females and 7 males), or pair‐fed (*n* = 6 females and 6 males; Table S1) were diluted 1:4, while lung homogenates heat‐exposed (*n* = 8 females and 7 males), control (*n* = 8 females and 7 males), or pair‐fed (*n* = 6 females and 6 males; Table S1) were diluted 1:5 in their respective diluents before analysis.

### 2.3 Microbiome analysis

DNA was extracted from each cecum sample (heat‐exposed (*n* = 8 females and 7 males), control (*n* = 8 females and 7 males), or pair‐fed (*n* = 8 females and 6 males)) using the DNeasy PowerLyzer PowerSoil Kit (Qiagen, Germantown, MD, USA; cat. no. 12855‐50) and then sequenced on the Illumina MiSeq platform (Illumina, San Diego, CA, USA). Downstream analyses of fastq files generated amplicon sequence variants (ASVs), which were analyzed using MicrobiomeAnalyst (Chong et al., 2020; Dhariwal et al., 2017). A full description of the microbiome analysis is provided in the Supporting Information S1.

### 2.4 RNA sequencing (RNASeq) and analysis

Total RNA was isolated using the RNeasy Mini Kit (Qiagen; cat. no. 74104) from the left lung lobe (heat‐exposed (*n* = 8 females and 7 males), control (*n* = 8 females and 7 males), or pair‐fed (*n* = 6 females and 6 males)) following the manufacturer's instructions and submitted to Novogene (Sacramento, CA, USA) for RNA sequencing using the NovaSeq 6000 PE150 platform. Differential expression analysis was performed with DESeq2 (version 1.38.3) and differentially expressed genes were visualized with principal component analysis (PCA) and volcano plots. Pathway analysis was conducted with iPathwayGuide (Advaita Corporation, Ann Arbor, Michigan, USA) (Bullert et al., 2024). A full description of RNASeq analysis can be found in the Supporting Information S1.

### 2.5 Serum metabolomics LC–MS instrument parameters

Serum metabolomics analyses (heat‐exposed (*n* = 8 females and 5 males), control (*n* = 7 females and 5 males), or pair‐fed (*n* = 4 females and 6 males)) were performed at the Northwest Metabolomics Research Center (University of Washington, Seattle, WA, USA) (Meador et al., 2020); see the Supporting Information S1 for additional details. Metabolomics data were analyzed with the MetaboAnalyst 6.0 online platform (Pang et al., 2021), including the PLS‐DA, heatmap, random forest analysis, KEGG enrichment pathway analysis, and one‐way ANOVA. All reported p‐values are adjusted p‐values based on MetaboAnalyst ANOVA analyses.

### 2.6 Network analysis

Integrated network analyses were performed for males and females using xMWAS (v. 1.0) (Uppal et al., 2018). These analyses were used to explore relationships between lung gene expression, serum metabolites, serum cytokines, and lung cytokines across exposure groups (heat‐exposed vs. control, heat‐exposed vs. pair‐fed, and pair‐fed vs. control). A full description of the correlation analyses is provided in the Supporting Information S1.

### 2.7 Statistical analysis

Body and tissue weights are reported as mean ± standard deviation. Comparisons between all groups for each respective cytokine were performed by a 2‐way ANOVA with Tukey multiple comparisons using GraphPad Prism (GraphPad Software, Boston, MA, USA; RRID:SCR_002798). Log fold change and *p*‐values based on a Mann–Whitney *U* test were calculated for ASV data, specifically for features that were statistically significant according to random forest analysis (mean decrease in accuracy (MDA) >0.01).

## 3 RESULTS

### 3.1 General responses to heat exposure

We first evaluated body and organ weights, as well as feed and water consumption, in heat‐exposed, pair‐fed, and control mice to determine whether gradual heat exposure induced gross physiological changes. Body and organ weights did not differ significantly between experimental groups (Figure S1a, Table S2). Feed consumption of heat‐exposed males and females compared to controls and heat‐exposed males compared to pair‐fed was not significantly different; however, feed consumption trended to be decreased in pair‐fed compared to heat‐exposed females (Figure S1b). Heat‐exposed mice showed significantly higher water consumption than their control and pair‐fed counterparts (Figure S1c).

### 3.2 Cytokine and chemokine alterations following heat exposure

Exposure to heat can trigger systemic inflammation and disrupt immune function, as evidenced by increased circulating cytokine and chemokine levels (Cantet et al., 2021; Chauhan et al., 2017; Di Vincenzo et al., 2024). Therefore, we assessed cytokine and chemokine levels in lung tissue and serum to characterize pulmonary and systemic inflammatory responses induced by heat exposure.

### 3.3 Lung cytokine and chemokine levels

Heat exposure significantly affected 3 out of 23 measured cytokines and chemokines in lung homogenates (Figure 1a–c). In males, IL‐17α levels were significantly lower in heat‐exposed animals than in pair‐fed animals. In females, IL‐17α levels did not differ significantly between experimental groups. MIP‐1α and MIP‐1β levels were significantly increased in females exposed to heat compared to both control and pair‐fed. In males, neither MIP‐1α nor MIP‐1β levels were significantly altered by heat exposure.

**FIGURE 1:**
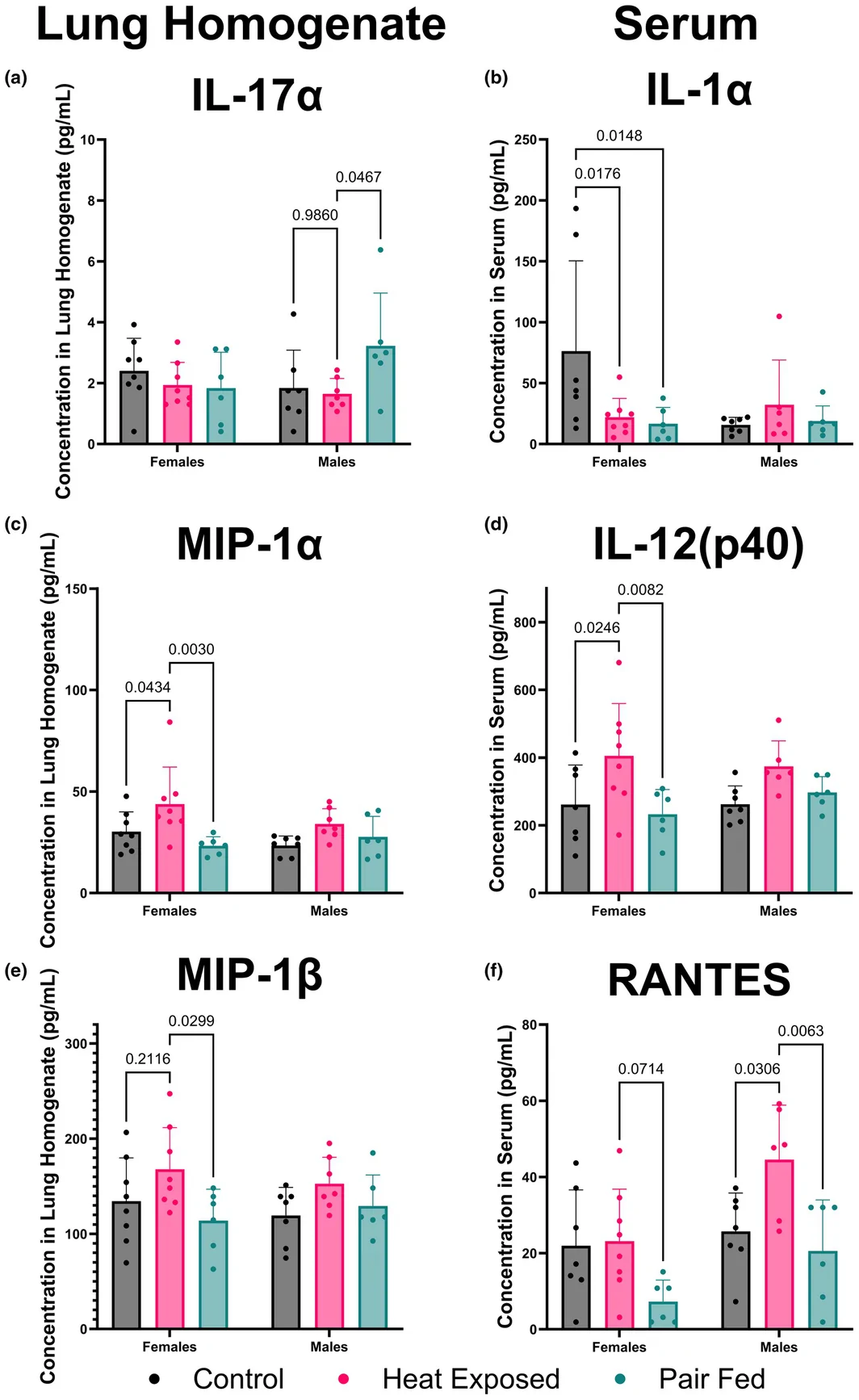
Heat exposure significantly affected the serum levels of (a) IL‐1α, (b) IL‐12(p40), and (c) RANTES, as well as the levels of (d) IL‐17α, (e) MIP‐1α, and (f) MIP‐1β in lung homogenate from male and female mice. A panel of 23 cytokines was measured using the Bio‐Plex Pro Mouse Cytokine 23‐Plex Assay, with no other significant effects of heat exposure observed on cytokine serum levels. Data are presented as mean ± standard deviation, and statistical significance was assessed using two‐way ANOVA with Tukey multiple comparisons. Lung homogenate *n* = 8 female control, female heat‐exposed; *n* = 6 female pair‐fed, male pair‐fed; *n* = 9 male control, male heat‐exposed. Serum *n* = 7 female control, male control; *n* = 8 female heat‐exposed, male pair‐fed; *n* = 6 female pair‐fed, male heat‐exposed.

### 3.4 Serum cytokine and chemokine levels

Heat exposure significantly altered 3 of 23 measured serum cytokines and chemokines (Figure 1d–f). IL‐1α levels were significantly decreased in heat‐exposed and pair‐fed females compared to controls, but were unaffected in males. IL‐12(p40) was significantly elevated in heat‐exposed females compared to pair‐fed animals and showed a trend toward higher levels than controls. Neither IL‐1α nor IL‐12(p40) levels differed significantly between experimental groups in males. RANTES levels were significantly increased in heat‐exposed males compared to both controls and pair‐fed, whereas a trend toward lower RANTES levels was observed in heat‐exposed versus pair‐fed females.

### 3.5 Microbiome changes following heat exposure

Earlier studies have demonstrated that abrupt changes in temperature alter the composition and function of the gut microbiome and the intestinal barrier (Hylander & Repasky, 2019; Wang, Wu, et al., 2024; Wen et al., 2021). In this study, we observed no significant changes in alpha diversity in females or males due to heat exposure (Figure S2a,b). Heat exposure also did not affect the beta diversity of microbial populations compared with control or pair‐fed females or males (Figure S2c,d). A random forest analysis identified 20 taxa (four phyla, five classes, five orders, three families, two genera, and one species) that differentiated the experimental groups (Figure 2). The Mann–Whitney *U* test showed that heat exposure did not significantly affect the relative abundances of these taxa.

**FIGURE 2:**
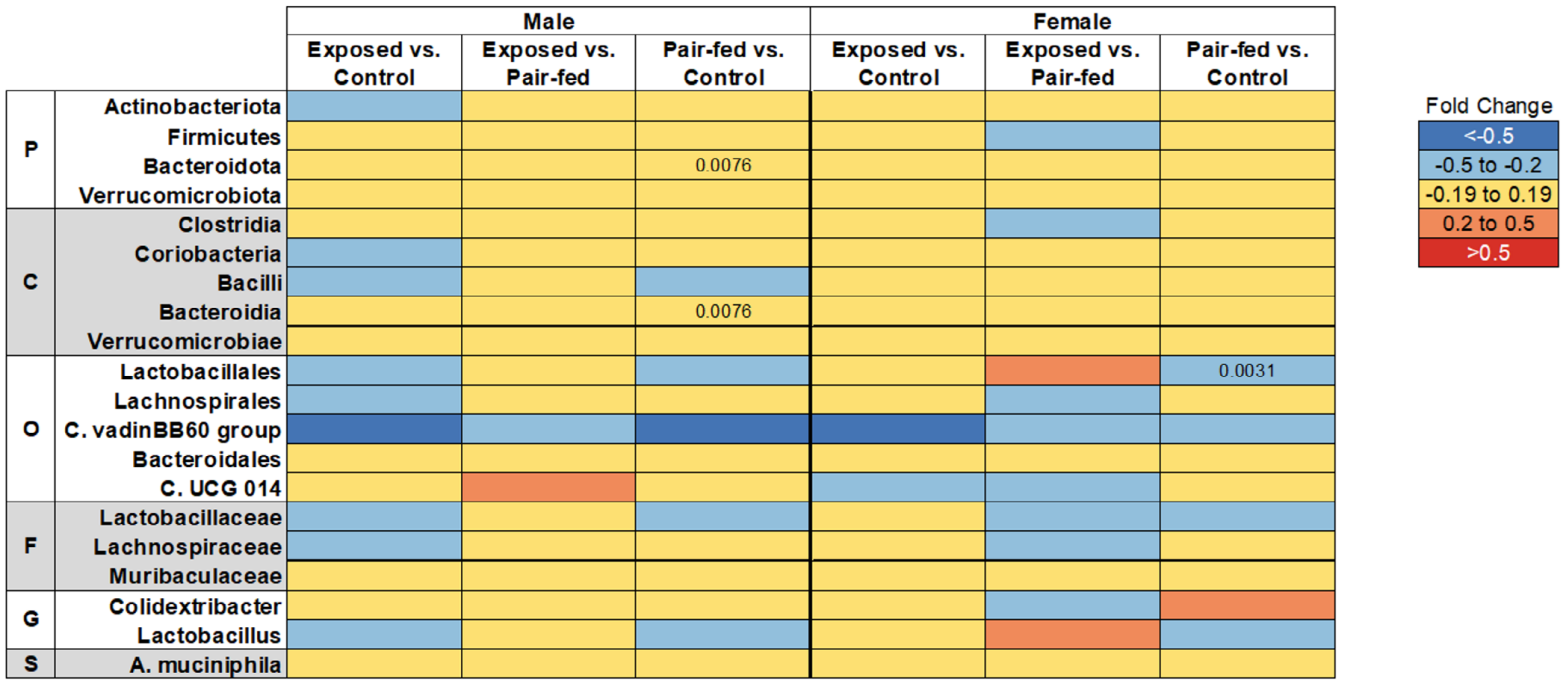
Random forest analysis identified 20 bacterial taxa important for distinguishing between experimental groups, categorized at different taxonomic levels (P: Phylum; C: Class; O: Order; F: Family; G: Genus; S: Species). Fold changes in bacterial abundance between experimental groups are color‐coded. Significant differences in amplicon sequence variant (ASV) data were determined using *p*‐values from the Mann–Whitney *U* test; *n* = 8 across all comparisons.

### 3.6 Transcriptomic effects in lungs after heat exposure

Sudden heat exposure is a known cause of transcriptomic responses in the lung (Liu et al., 2020; Nan et al., 2023; Wang, Fan, et al., 2024). In the present study, PCA did not reveal a separation of gene expression profiles in the lungs of males or females by experimental groups (Figure S3). However, we observed significant transcriptomic alterations in the lung following heat exposure. Therefore, iPathwayGuide was performed to characterize biological processes affected by heat exposure in the lung. Heat exposure primarily affected biological processes related to immune activation compared with control and pair‐fed groups (Figures 4 and 5).

### 3.7 Biological processes altered in lungs from heat‐exposed versus control mice

In females, heat exposure resulted in upregulation of 90 genes and downregulation of 31 genes compared to controls (Figure 3a). Similarly, in males, 105 genes were upregulated, and 48 were downregulated relative to controls (Figure 4a). In the iPathwayGuide analysis, two immune‐related biological processes, “Immune System Process” and “Lymphocyte Activation,” were significantly altered in both heat‐exposed females and males compared to controls (Figures 3c and 4c). “Immunoglobulin‐Mediated Immune Response,” “B Cell‐Mediated Immunity,” and “Immune Response” were altered only in heat‐exposed females compared to controls (Figure 3c). Twenty‐two genes were associated with these biological processes, with 10 genes upregulated and 12 downregulated in females following heat exposure. In males, heat exposure affected additional immune‐related processes; for example, “Leukocyte Activation,” “Cell Activation,” and “B Cell Activation.” Sixteen genes were associated with these biological processes, with 13 genes significantly upregulated and 3 downregulated in heat‐exposed males.

**FIGURE 3:**
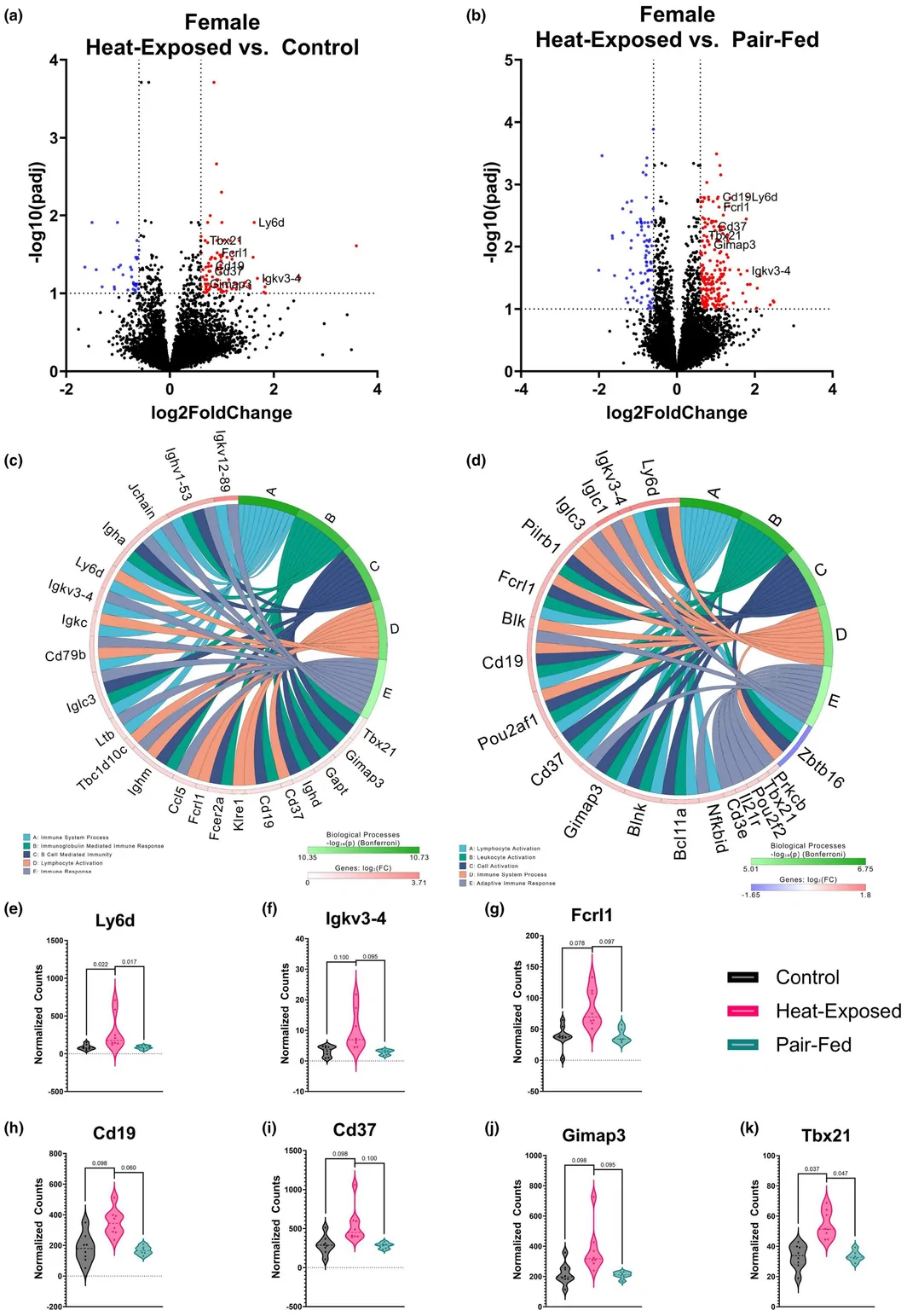
RNA sequencing analysis of lung tissue from heat‐exposed female mice revealed significant gene expression changes and disrupted biological processes compared to control and pair‐fed mice. Volcano plots illustrate differentially expressed genes (DEGs) in (a) heat‐exposed versus control and (b) heat‐exposed versus pair‐fed, based on thresholds of *p*‐value <0.1 and |log fold change| >0.3. Dotted lines indicate the DEG selection thresholds. Chord diagrams illustrate the association between DEGs and biological processes, showing the top five biological processes linked to 10 genes per process in (c) heat‐exposed versus control and (d) heat‐exposed versus pair‐fed. Chord diagrams were generated using Advaita Corporation iPathwayGuide. Expression patterns of selected DEGs shared across altered biological processes are shown in panels (e–k). DEG normalized counts are presented as mean ± standard deviation. Control and heat‐exposed *n* = 8; pair‐fed *n* = 6.

**FIGURE 4:**
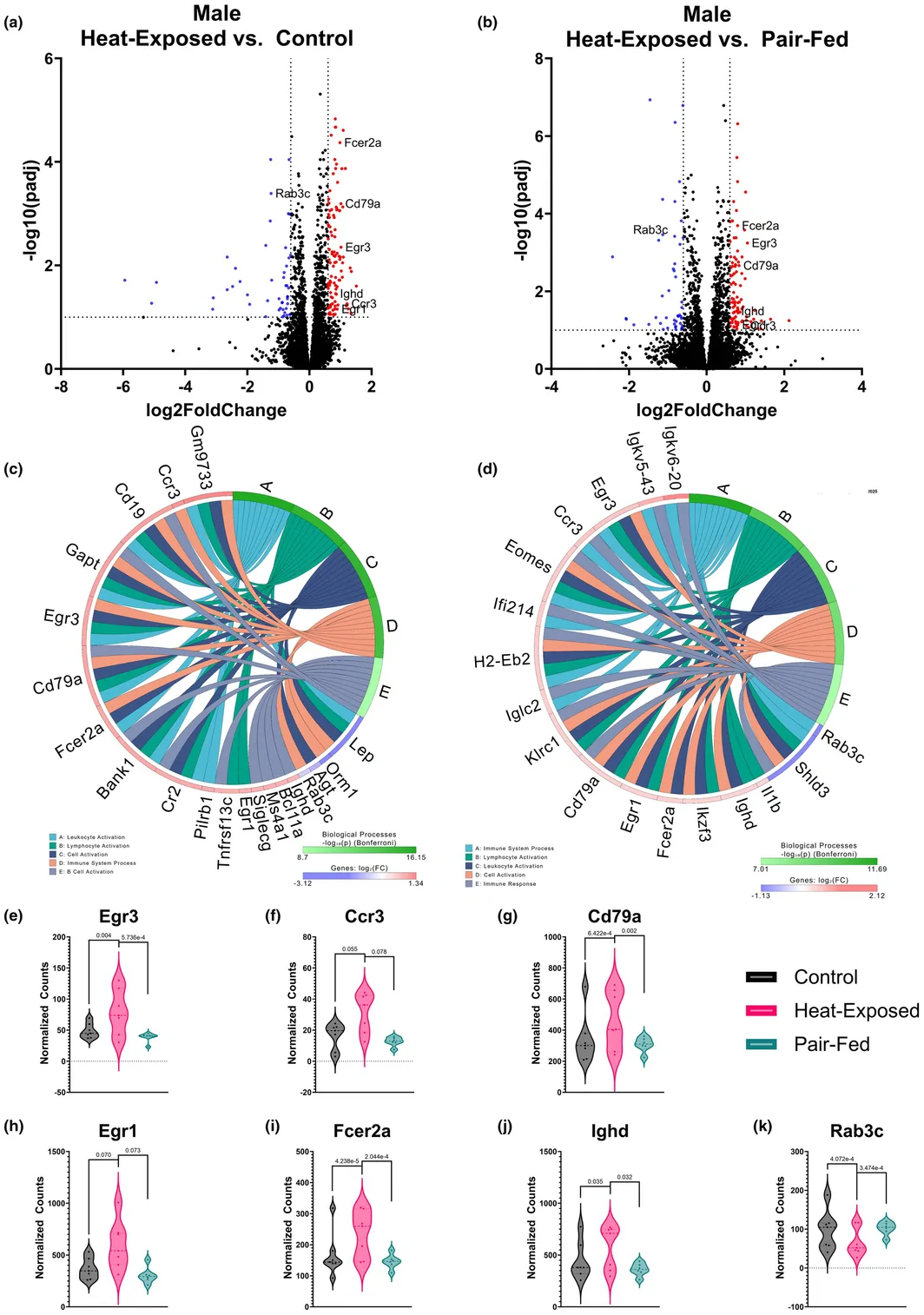
RNA sequencing analysis of lung tissue from heat‐exposed male mice revealed significant gene expression changes and disrupted biological processes compared to control and pair‐fed mice. Volcano plots display differentially expressed genes (DEGs) in (a) heat‐exposed versus control and (b) heat‐exposed versus pair‐fed, based on thresholds of *p*‐value <0.1 and |log fold change| >0.3. The dotted lines indicate the DEG selection thresholds. Chord diagrams illustrate the association between DEGs and biological processes, showing the top five biological processes linked to 10 genes per process in (c) heat‐exposed versus control and (d) heat‐exposed versus pair‐fed. Chord diagrams were generated using Advaita Corporation iPathwayGuide. Expression patterns of selected DEGs shared across altered biological processes are shown in panels (e–k). DEG normalized counts are presented as mean ± standard deviation; *n* = 7 across all comparisons.

Four genes were upregulated in both male and female heat‐exposed versus controls, including Fc fragment of IgE receptor II (*Fcer2a*), CD19 molecule (*Cd19*), Immunoglobulin heavy constant delta (*Ighd*), and GRB2‐binding adaptor protein, transmembrane (*Gapt*) (Figure S4). *Fcer2a* was associated with “Immunoglobulin Mediated Immune Response” and “B Cell‐Mediated Immunity” in females and “Leukocyte Activation,” “Cell Activation,” and “Immune System Process” in males. *Fcer2a* modulates immune responses in the lung and is expressed on various lung immune cells, including B cells and macrophages (Cernadas et al., 1999). *Cd19*, which positively regulates B cell activation (Karnell et al., 2014), was associated with “Immunoglobulin Mediated Immune Response,” “B Cell‐Mediated Immunity,” and “Lymphocyte Activation” in females and the top five biological processes in males. *Ighd*, which is involved in B cell receptor signaling and adaptive immune response (Schroeder Jr. & Cavacini, 2010), was associated with “Immunoglobulin Mediated Immune Response” and “B Cell Mediated Immunity” in females and “B Cell Activation” in males. Finally, *Gapt*, which plays an important role in B cell activation (Liu & Zhang, 2008), was associated with “Immunoglobulin Mediated Immune Response” and “B Cell‐Mediated Immunity” in females and the top five biological processes in males.

### 3.8 Biological processes altered in the lungs of heat‐exposed versus pair‐fed mice

Compared with pair‐fed animals, heat exposure upregulated 91 genes and downregulated 46 in females (Figure 3b), and upregulated 207 genes and downregulated 80 in males (Figure 4b). In the iPathwayGuide analysis, several immune‐related biological processes, including “Immune System Process,” “Cell Activation,” “Leukocyte Activation,” and “Lymphocyte Activation,” were significantly altered in heat‐exposed females and males compared to pair‐fed (Figures 3d and 4d). In addition, “Immune System Process” and “Adaptive Immune Response” were altered in heat‐exposed versus pair‐fed females (Figure 3d), with 18 genes significantly elevated and 1 significantly decreased in these biological processes. In male heat‐exposed versus pair‐fed mice, “Cell Activation” and “Immune Response” were altered (Figure 4d). Fifteen significantly elevated and 2 significantly decreased genes in heat‐exposed versus pair‐fed males were associated with these biological processes. No shared DEGs were detected between males and females, despite overlapping biological processes.

### 3.9 Shared lung transcriptomic responses to heat exposure

Shared effects of heat exposure on the lung transcriptome, compared with control and pair‐fed females, likely indicate that the effects are mediated by heat rather than reduced feed intake. Heat exposure altered two biological processes, “Immune System Process” and “Lymphocyte Activation,” compared with control and pair‐fed females. These processes were associated with seven shared DEGs: lymphocyte antigen 6 complex, locus D (*Ly6d*), immunoglobulin kappa variable 3–4 (*Igkv3‐4*), Fc receptor‐like 1 (*Fcrl1*), CD19 molecule (*Cd19*), CD37 molecule (*Cd37*), GTPase, IMAP family member 3 (*Gimap3*), and T‐box transcription factor 21 (*Tbx21*) (Figure 3e–k). In males, heat exposure altered four biological processes, “Immune System Process,” “Lymphocyte Activation,” “Leukocyte Activation,” and “Cell Activation,” compared to both control and pair‐fed animals, with seven shared DEGs: early growth response 3 (*Egr3*), C‐C motif chemokine receptor 3 (*Ccr3*), CD79a molecule (*Cd79a*), early growth response 1 (*Egr1*), Fc fragment of IgE receptor II (*Fcer2a*), immunoglobulin heavy constant delta (*Ighd*), and RAB3C, member RAS oncogene family (*Rab3c*) (Figure 4e–k). Across both sexes, heat exposure affected transcripts annotated to immune‐related pathways, including genes associated with B‐cell markers (*Cd19*, *Cd37*, *Cd79a*, *Fcrl1*), immunoglobulin components (*Ighd*, *Igkv3‐4*), transcriptional regulators (*Egr1*, *Egr3*, *Tbx21*), and chemokine or signaling pathways (*Ccr3*, *Gimap3*, *Rab3c*) in the lung.

### 3.10 Serum metabolome alterations following heat exposure

Serum metabolomics was performed to identify responses in serum metabolites related to heat exposure. PLS‐DA plots of females and males (Figures 5a and 6a) and heatmaps illustrating the top 25 metabolites most affected by heat exposure (Figures 5b and 6b) showed a separation between heat‐exposed and control groups, with the pair‐fed group overlapping the other two experimental groups in the PLS‐DA. The random forest analysis identified N‐Ac‐glutamate, pseudouridine, and N‐Ac‐alanine in females and N‐Ac‐phenylalanine and ornithine in males as candidate discriminating metabolites with an MDA score >0.01 (Figures 5c and 6c). These metabolites were identified in both the random forest analysis and the heatmaps; however, none differed significantly based on ANOVA. Based on KEGG enrichment analysis, heat exposure affected eight KEGG pathways in females (Figure 5d) and 20 pathways in males (Figure 6d). Among enriched pathways, “Glycine, Serine, and Threonine Metabolism” was affected by heat exposure in both sexes (Figures 5e and 6e), with a significant increase in threonine and decrease in cystathionine due to heat exposure compared to pair‐fed in females only. Overall, metabolomics analyses revealed only small alterations in endogenous metabolites in both males and females compared with their respective controls and pair‐fed controls.

**FIGURE 5:**
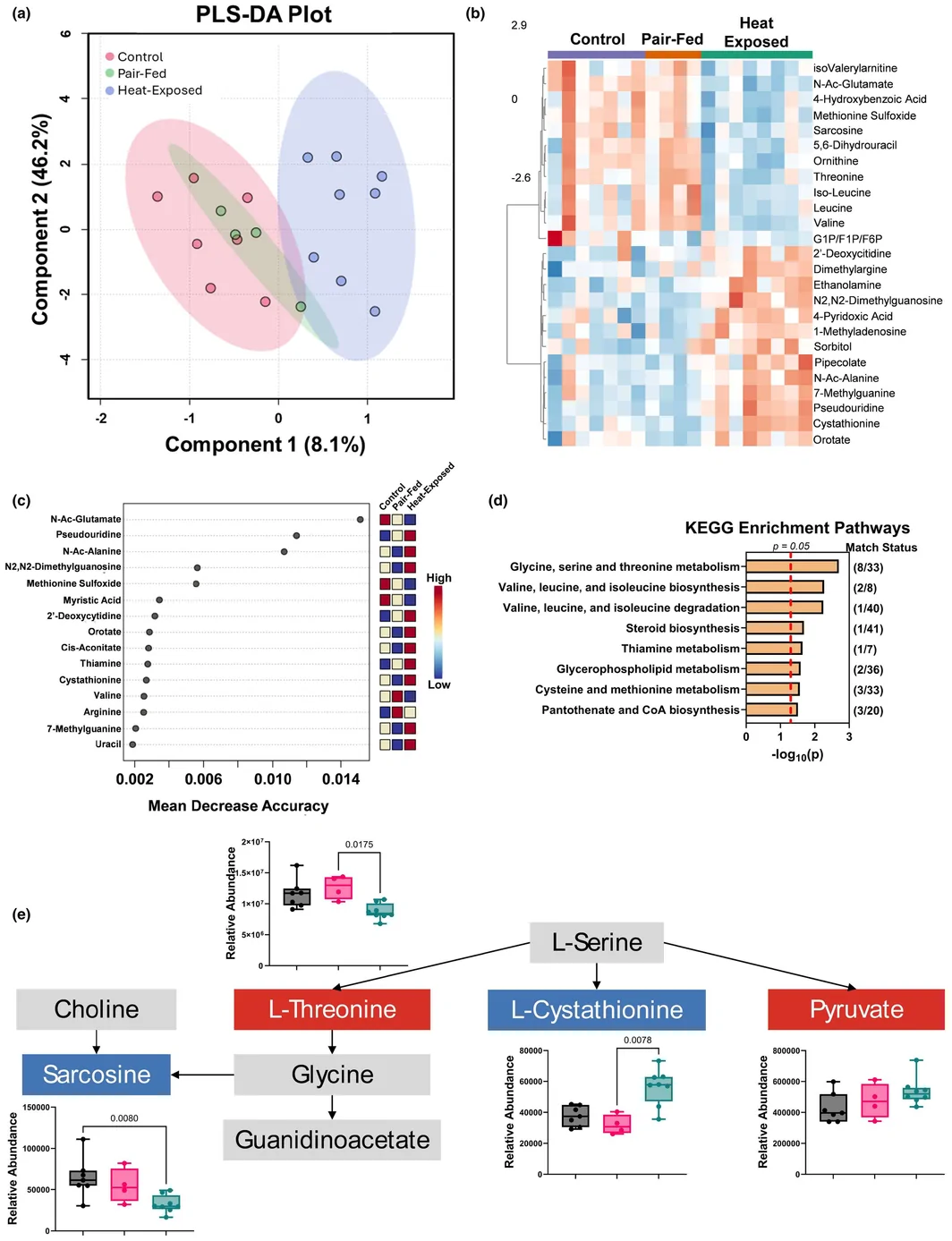
Heat exposure altered the serum metabolome in female mice, as shown by (a) partial least squares discriminant analysis (PLS‐DA), with (b) the top 25 metabolites shown in a heatmap. (c) Random forest analysis identified three key metabolites that distinguished experimental groups. (d) Heat exposure also significantly impacted eight KEGG pathways, including (e) the ‘Glycine, Serine, and Threonine Metabolism’ pathway. Red indicates increased and blue indicates decreased metabolite levels. Metabolite levels are plotted as mean ± standard deviation; *n* = 7 control (black), *n* = 4 heat‐exposed (pink), *n* = 8 pair‐fed (green).

**FIGURE 6:**
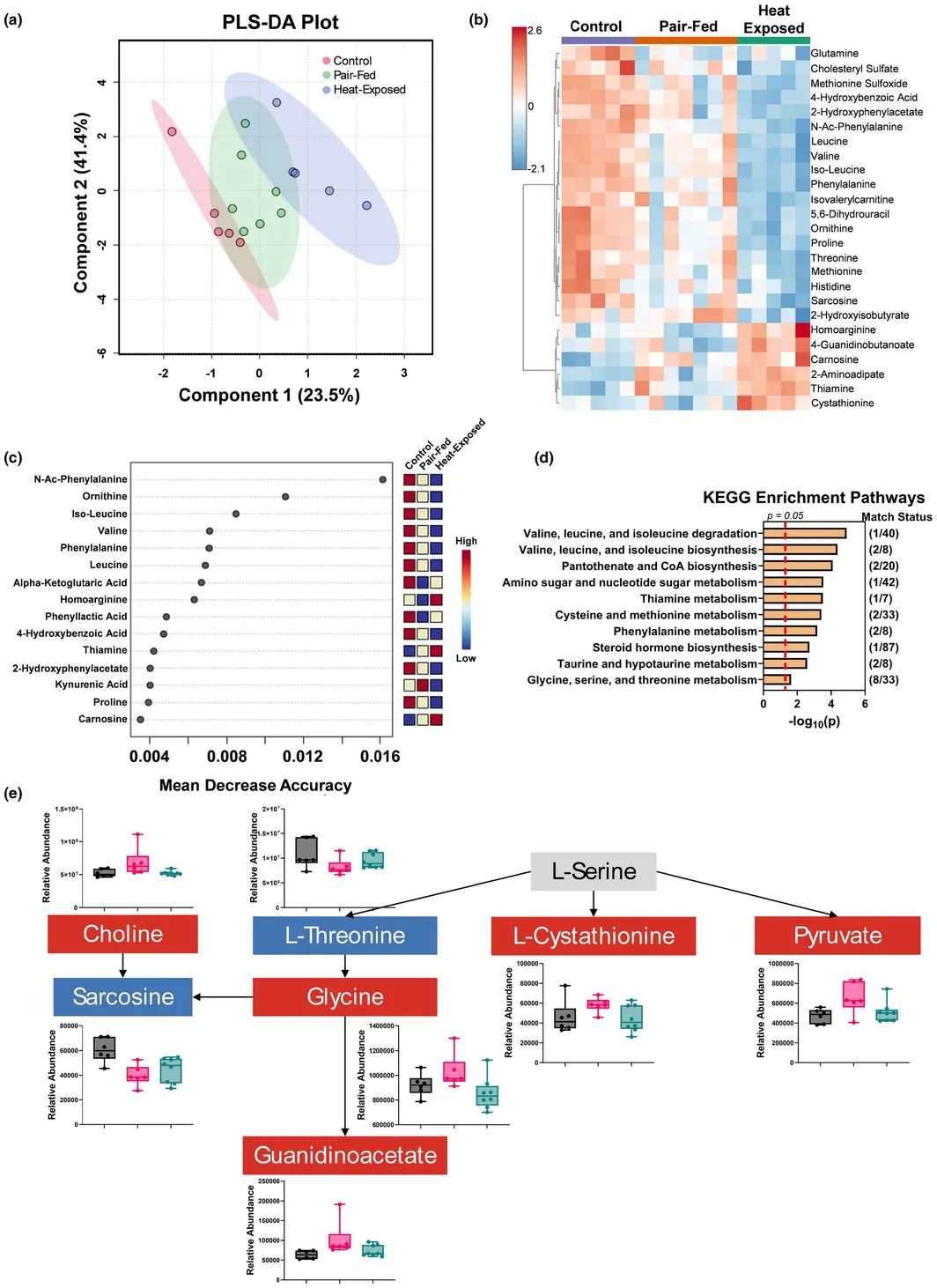
Heat exposure altered the serum metabolome in male mice, as shown by (a) partial least squares discriminant analysis (PLS‐DA), with (b) the top 25 metabolites shown in a heatmap. (c) Random forest analysis identified key metabolites that distinguished experimental groups. (d) Heat exposure also significantly altered several KEGG pathways, including (e) the ‘Glycine, Serine, and Threonine Metabolism’ pathway. Red indicates increased and blue indicates decreased metabolite levels. Metabolite levels are plotted as mean ± standard deviation; *n* = 5 control (black), *n* = 7 heat‐exposed (pink), *n* = 5 pair‐fed (green).

### 3.11 Network analysis

Correlation‐based network analyses were performed using xMWAS (Uppal et al., 2018) to integrate lung transcriptomic profiles with serum metabolites, serum cytokines, and lung cytokines, thereby characterizing systemic and pulmonary immunometabolic associations in heat‐exposed female and male mice (Figure 7). Networks were visualized for three pairwise comparisons: heat‐exposed versus control (Figure 7a,b), heat‐exposed versus pair‐fed (Figure 7c,d), and pair‐fed versus control (Figure 7e,f).

**FIGURE 7:**
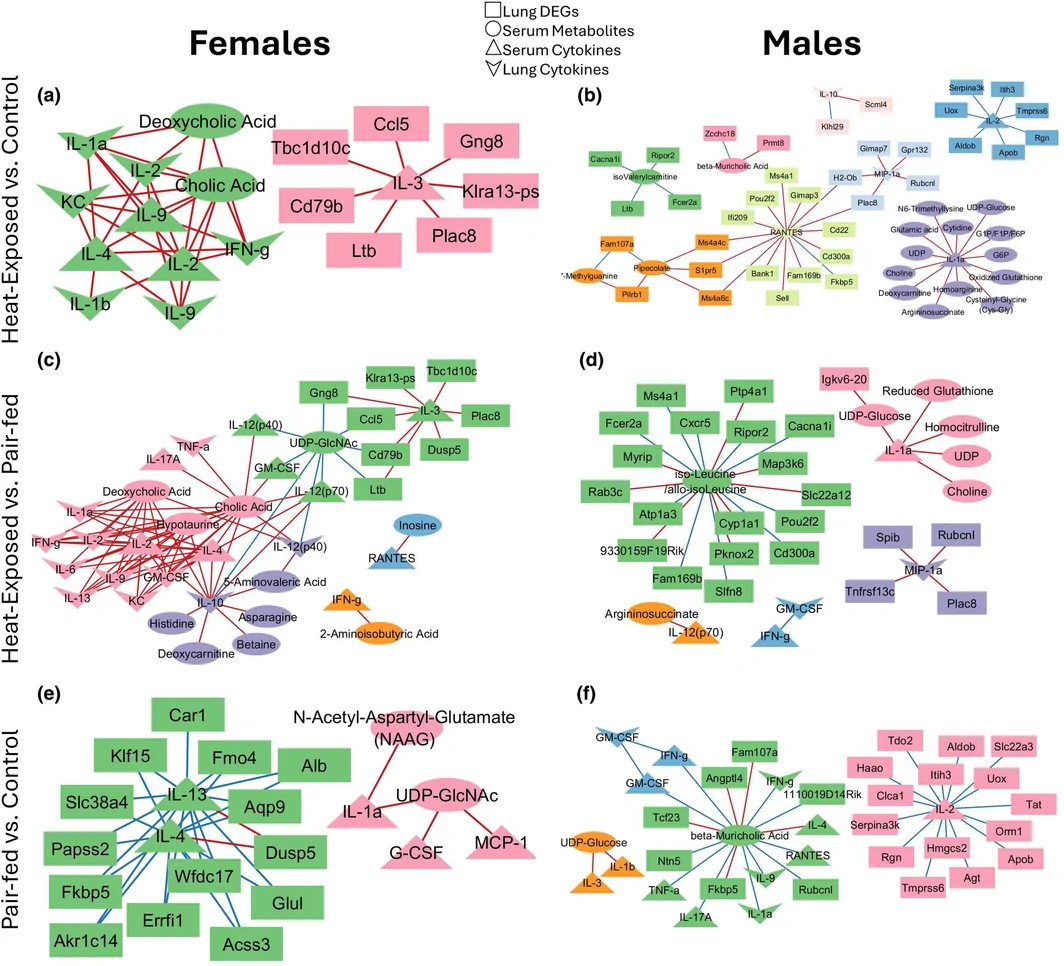
Network analyses showing associations in females and males comparing lung differentially expressed genes (DEGs, rectangles), serum metabolites (ellipses), serum cytokines (triangles), and lung cytokines (v‐shape) in heat‐exposed versus control (a, b), heat‐exposed versus pair‐fed (c, d), and pair‐fed versus control (e, f). Line colors indicate the direction of correlations, with blue representing negative and red representing positive correlations. The color coding of genes, cytokines, and metabolites reflects clusters identified by xMWAS (Uppal et al., 2018); comparisons for females had an *n* = 7 control, *n* = 4 pair‐fed, *n* = 8 heat‐exposed while males had *n* = 5 control, *n* = 7 pair‐fed, *n* = 5 heat‐exposed.

### 3.12 Heat‐exposed versus control correlation networks

In females (Figure 7a), the network comprised two distinct clusters containing seven DEGs, two serum metabolites (cholic acid and deoxycholic acid), four serum cytokines, and six lung cytokines, with all 36 correlations being positive. Lung IL‐1α formed a central hub of the first cluster and was positively correlated with serum IL‐4, serum IL‐9, serum IL‐2, and both bile acids. A second cluster was organized around serum IL‐3, which was positively correlated with seven DEGs.

In males (Figure 7b), the network was more complex, comprising eight clusters with 35 DEGs, 17 serum metabolites, two serum cytokines, and three lung cytokines, totaling 39 positive and 16 negative correlations. Lung IL‐10, lung MIP‐1α, lung RANTES, serum IL‐1α, and serum IL‐2 emerged as central hubs. Lung MIP‐1α was positively correlated with five DEGs; lung RANTES was positively associated with 14 DEGs and negatively with the DEG *Fkbp5* (FK506 binding protein 5); and serum IL‐1α was positively correlated with 13 serum metabolites. Serum IL‐2 formed an additional hub, negatively correlated with seven DEGs. The serum metabolite isovalerylcarnitine was positively correlated with four DEGs. Three smaller clusters were also identified: a lung IL‐10 cluster, in which IL‐10 was correlated with the DEGs *Scml4* (Scm Polycomb Group Protein Like 4) and *Klhl29* (kelch like family member 29); a cluster centered on the serum metabolite β‐muricholic acid, correlated with the DEGs *Zcchc18* (zinc finger CCHC‐type containing 18) and *Prmt8* (Protein Arginine Methyltransferase 8); and a cluster centered on the serum metabolite pipecolate, correlated with five DEGs.

Two DEGs, *Plac8* (Placenta Associated 8) and *Ltb* (Lymphotoxin Beta), appeared in both the female and male networks: in females, both were positively correlated with serum IL‐3; in males, *Plac8* was positively correlated with lung MIP‐1α and lung RANTES, whereas, as stated above, *Ltb* was negatively correlated with isovalerylcarnitine.

### 3.13 Heat‐exposed versus pair‐fed correlation networks

In females (Figure 7c), the network comprised five clusters, including eight DEGs, 11 serum metabolites, 9 serum cytokines, and 11 lung cytokines, yielding 72 positive and 9 negative correlations. The largest cluster was centered on the serum metabolites cholic acid, deoxycholic acid, and hypotaurine and included the lung cytokines GM‐CSF, IFN‐γ, IL‐1α, IL‐2, IL‐6, IL‐9, IL‐13, KC, and TNF‐α, along with the serum cytokines IL‐4 and IL‐17A, all positively interconnected. The second cluster was organized around the serum metabolite UDP‐GlcNAc as its central hub, which was negatively correlated with three serum cytokines, four DEGs, and two lung cytokines. Within the same cluster, serum IL‐3 formed a secondary hub linked by positive correlations to eight DEGs. A third cluster was organized around lung IL‐10 and lung IL‐12(p40) and correlated with serum metabolites 5‐aminovaleric acid, asparagine, betaine, deoxycarnitine, and histidine. Two smaller clusters comprised serum RANTES paired with the serum metabolite inosine, and serum IFN‐γ paired with the serum metabolite 2‐aminoisobutyric acid.

In males (Figure 7d), the network comprised five clusters with 23 DEGs, seven serum metabolites, three serum cytokines, and two lung cytokines, with 19 positive and 11 negative correlations. The largest cluster was centered on the serum metabolite iso‐leucine/allo‐isoleucine, which was correlated with 18 DEGs, with both positive and negative correlations. A second cluster was anchored by serum IL‐1α, which was positively correlated with five serum metabolites (choline, homocitrulline, reduced glutathione, UDP, and UDP‐glucose) and one DEG (*Igkv6‐20* (immunoglobulin kappa variable 6‐20)). A third cluster contained lung MIP‐1α, which was positively correlated with four DEGs (*Plac8, Rubcnl* (rubicon like autophagy enhancer), *Spib* (Spi‐B transcription factor), and *Tnfrsf13c* (TNF receptor superfamily member 13C)). Two smaller clusters comprised serum IL‐12(p70) paired with the serum metabolite argininosuccinate, and lung GM‐CSF paired with serum IFN‐γ. *Plac8* had hub status across both sexes and was positively correlated with serum IL‐3 in females and with lung MIP‐1α in males, mirroring the heat‐exposed versus control comparison above. *Ltb* was observed in the female network, with a positive association with IL‐3.

### 3.14 Pair‐fed versus control correlation networks

In females (Figure 7e), the network included 14 DEGs, two serum metabolites, and five serum cytokines distributed across two clusters, with 6 positive and 25 negative correlations. All five cytokines in this network were measured in serum. The first cluster was anchored by a dual hub of serum IL‐13 and serum IL‐4, each of which was predominantly negatively correlated with 14 DEGs. The second cluster contained N‐acetyl‐aspartyl‐glutamate (NAAG), which was positively correlated with serum IL‐1α, and UDP‐GlcNAc, which was positively correlated with serum G‐CSF, serum MCP‐1, and serum IL‐1α.

In males (Figure 7f), the network comprised 22 DEGs, two serum metabolites, nine serum cytokines, and four lung cytokines across four clusters, with 7 positive and 28 negative correlations. The largest cluster was centered on the serum metabolite β‐muricholic acid. Serum IL‐4 was the only cytokine positively correlated with this bile acid, whereas the serum cytokines IL‐17A, RANTES, and TNF‐α, and the lung cytokines IFN‐γ, IL‐9, and IL‐1α were all negatively correlated with this endogenous metabolite. The cluster also contained seven DEGs positively or negatively associated with β‐muricholic acid. A second cluster was anchored by serum IL‐2 and was negatively correlated with 15 DEGs. A third cluster comprised lung and serum GM‐CSF and serum IFN‐γ, while a fourth cluster contained serum IL‐1β and serum IL‐3 linked through the serum metabolite UDP‐glucose. Serum IL‐4 appeared in both the female and male pair‐fed versus control networks, negatively correlated with DEGs in females, and positively correlated with β‐muricholic acid in males.

## 4 DISCUSSION

### 4.1 General responses to heat exposure

In studies of sudden heat exposure, animals typically suppress feed intake to reduce the thermogenic burden of digestion (Baumgard & Rhoads, 2013; Roach et al., 2024; Xiao et al., 2020). In contrast, the gradual heat‐exposure paradigm used here did not significantly alter feed consumption compared with controls, suggesting that thermal adaptation in this model occurs without compensatory hypophagia. The trend toward decreased feed consumption in pair‐fed females limits the heat‐versus‐pair‐fed comparison. Heat‐exposed mice consumed significantly more water than control and pair‐fed mice, likely to maintain fluid balance during heat exposure.

### 4.2 Cytokine and chemokine alterations following heat exposure in the lungs and serum

Heat exposure can trigger systemic inflammation and disrupt immune function, as evidenced by altered circulating cytokine and chemokine levels (Cantet et al., 2021; Chauhan et al., 2017; Di Vincenzo et al., 2024). In the present study, gradual heat exposure affected several cytokines and chemokines in the lung in heat‐exposed male and female mice. Reduced IL‐17α in males and elevated MIP‐1α and MIP‐1β in females distinguished heat‐exposed mice from pair‐fed groups. The decrease in lung IL‐17α in males may reflect impaired mucosal defense or a regulatory dampening of IL‐17‐driven tissue stress, as IL‐17α plays a crucial role in maintaining mucosal immunity (Gurczynski & Moore, 2018; McGeachy et al., 2019) and in supporting health during injury response, physiological stress, or infection (Sun et al., 2023; Veldhoen, 2017). Elevated MIP‐1α and MIP‐1β in the lungs of females suggest increased immune cell recruitment and activation (Lillard et al., 2003), consistent with an inflammatory response during heat exposure (Cantet et al., 2021; Leon & Helwig, 2010).

We also observed significant heat‐mediated effects on several cytokines and chemokines in the serum, including increased IL‐12(p40) in females and RANTES in males. These elevated serum IL‐12(p40) levels in heat‐exposed females indicate a strong immune response that drives Th1 and Th2 responses (Trinchieri, 2003). Increased serum RANTES levels suggest heightened recruitment of immune cells, a hallmark of chronic inflammation or immune activation (Ajuebor et al., 2001; Koya et al., 2006; Machura et al., 2024; Zeng et al., 2022). In contrast, IL‐1α was altered in both heat‐exposed and pair‐fed females relative to controls. Because the pair‐feeding paradigm used here did not produce a statistically significant reduction in feed intake, this shared decrease cannot be confidently attributed to caloric restriction. It may instead reflect non‐caloric features common to both manipulations such as HPA‐axis activation from the feed‐restriction procedure itself, or a circadian/feeding‐pattern effect of timed feed delivery in pair‐fed animals (Ellacott et al., 2010; Manoogian & Panda, 2017). Together this modest serum cytokine signature (3 of 23 altered) suggests a limited rather than systemic inflammatory response at this time point.

Notably, chemokines that were increased after heat exposure in the lung, such as MIP‐1α and MIP‐1β, and in serum, such as RANTES, belong to the CC chemokine subfamily and are known for their roles in T cell adhesion and migration (Zeng et al., 2022). Because most of the 23 serum cytokines and chemokines we measured were unaffected, a single gradual heat exposure produced only a limited systemic inflammatory signal at the sampled time point. Whether repeated insults, as occur during multi‐day heat waves, would allow incomplete resolution of inflammation to lead to sustained, low‐grade inflammation remains an important open question.

### 4.3 Microbiome changes following heat exposure

We examined changes in the cecal microbiota after heat exposure because the microbiome influences inflammatory markers throughout the body (Di Vincenzo et al., 2024; Haase et al., 2018; Wen & Duffy, 2017). Prior studies have shown that abrupt temperature changes alter the composition and function of the gut microbiome and the intestinal barrier (Hylander & Repasky, 2019; Wang, Wu, et al., 2024; Wen et al., 2021). In the gradual exposure paradigm used in this study, alpha and beta diversity did not change in either females or males. A random forest analysis identified several taxa that differentiated the experimental groups in male and female mice; however, univariate testing (Mann–Whitney *U* test) revealed no statistically significant difference between heat‐exposed and control or pair‐fed animals. This suggests that the gradual heat‐exposure paradigm used in this study has a less pronounced effect on the microbiome than the abrupt heat‐exposure paradigms used in the earlier studies.

### 4.4 Transcriptomic effects in lungs after heat exposure

Sudden heat exposure is a known cause of transcriptomic responses in the lung (Liu et al., 2020; Nan et al., 2023; Wang, Fan, et al., 2024). In our study, heat exposure primarily affected biological processes related to immune activation compared with control and pair‐fed groups, consistent with the cytokine and chemokine data discussed above. Both sexes showed altered “Immune System Process” and “Lymphocyte Activation” pathways. Females also exhibited changes in “Immunoglobulin‐Mediated Immune Response,” “B Cell‐Mediated Immunity,” and “Immune Response,” while males showed additional alterations in “Leukocyte Activation,” “Cell Activation,” and “B Cell Activation.”

These pathway‐level differences were reflected in individual genes. In females, the shared “Immune System Process” and “Lymphocyte Activation” alterations across control and pair‐fed comparisons were driven by *Cd19*, *Cd37*, *Fcrl1*, *Gimap3*, *Igkv3‐4*, *Ly6d*, and *Tbx21*. In males, these same two processes, together with “Leukocyte Activation” and “Cell Activation,” were associated with *Ccr3*, *Cd79a*, *Egr1*, *Egr3*, *Fcer2a*, *Ighd*, and *Rab3c*. Collectively, these genes govern B‐cell activation (*Cd19*, *Cd37*, *Cd79a*, *Fcrl1*), immunoglobulin production (*Igkv3‐4*, *Ighd*), and inflammatory signaling (*Egr1*) (Zou & Zeng, 2023), alongside transcription factors with broader stress‐response and cellular activation roles (*Egr3*, *Rab3c*, *Tbx21/T‐bet*), chemokine receptors (*Ccr3*), and survival‐associated genes (*Gimap3*) that contribute to immune cell recruitment and persistence (Conroy & Williams, 2001; Huang & Bi, 2021). These findings are consistent with previously documented roles of the lung in heat‐induced immune responses (Wheeler & Wong, 2007) and indicate that gradual heat exposure induces immune activation in the lung, with partially overlapping molecular signatures observed in male and female mice.

### 4.5 Serum metabolome alterations following heat exposure

Serum metabolomic analysis revealed only small, non‐significant shifts in endogenous metabolites in heat‐exposed mice of both sexes compared with control and pair‐fed groups. The random forest analysis identified N‐acetylglutamate, pseudouridine, and N‐acetylalanine in females and N‐acetyl‐phenylalanine and ornithine in males as altered by heat exposure. KEGG enrichment analysis indicated pathway disruptions following heat exposure, with the “Glycine, Serine, and Threonine Metabolism” pathway, which contributes to carbohydrate and energy utilization efficiency (Wu et al., 2021), affected in both male and female mice. Overall, heat exposure did not significantly alter any metabolites, including those identified by the random forest or pathway analyses. The absence of statistically significant changes in individual serum metabolites among control, heat‐exposed, and pair‐fed mice suggests that the modest reduction in feed intake observed in pair‐fed females did not exert a detectable effect on the systemic metabolome under the conditions of this study. The lack of robust systemic metabolomic responses contrasts with earlier work reporting significant alterations in the metabolome following heat exposure (Ippolito et al., 2014), suggesting that different heat‐exposure paradigms and time points may yield distinct metabolic profiles. Additional studies with larger sample sizes and targeted metabolomics measurements are needed to determine if the metabolites and pathways identified represent biologically meaningful responses to gradual heat exposures.

### 4.6 Network analysis

Multi‐omics integration with xMWAS was used to assess whether alterations in individual lung transcripts, serum metabolites, and dual‐compartment cytokines (lung and serum) following gradual heat exposure provide biologically interpretable patterns (Uppal et al., 2018) that can be explored further in future mechanistic studies. Because we examined heat‐exposed animals against both ad libitum and pair‐fed controls, the network analysis allowed us to identify immunometabolic signatures attributable to heat exposure rather than to HPA‐axis activation due to feed restriction or to a circadian/feeding‐pattern effect of timed feed delivery in pair‐fed animals (Ellacott et al., 2010; Manoogian & Panda, 2017).

Two upregulated genes, *Plac8* (Placenta‐specific 8) and *Ltb* (Lymphotoxin beta), were present in the heat‐exposed versus control networks in both male and female mice. In females, both genes were positively correlated with serum IL‐3. In males, *Plac8* was positively correlated with lung MIP‐1α and RANTES, while *Ltb* was negatively correlated with isovalerylcarnitine. *Plac8* was also a hub in the heat‐exposed versus pair‐fed networks in both sexes and was again positively correlated with serum IL‐3 in females and with lung MIP‐1α in males. Both genes have well‐defined immune roles consistent with a pulmonary inflammatory response: *Plac8* is expressed in the lung (Ledford et al., 2007), required for innate antibacterial defense (Ledford et al., 2007), modulates monocytes in sepsis in mice and humans (Zhang et al., 2024), and is expressed by activated T‐cell subsets (Slade et al., 2020; Tibbitt et al., 2019; Yang et al., 2021). *Ltb* encodes a subunit of the membrane lymphotoxin complex that signals through LTβR, which is critical for developing and maintaining secondary lymphoid organs and for regulating immune cell migration and homeostasis (Piao et al., 2021; Shou et al., 2021). These findings identify *Plac8* and *Ltb* as novel candidates for mechanistic follow‐up.

Lung GM‐CSF appeared in the heat‐exposed versus pair‐fed networks of both sexes. In females, GM‐CSF was positively correlated with cholic acid and negatively correlated with UDP‐GlcNAc, whereas in males it was negatively correlated with interferon‐γ (IFN‐γ). GM‐CSF is a pleiotropic cytokine that drives myeloid cell production and function and shapes T‐cell activation (Bhattacharya et al., 2015). Like IL‐3, which was positively correlated with *Plac8* and *Ltb* in females, GM‐CSF is a common β‐receptor‐signaling cytokine (Shearer et al., 2003). Because both IL‐3 and GM‐CSF are cytokines that activate innate immune responses, particularly monocytes and neutrophils (Heled et al., 2013; Oki et al., 2022), the identification of IL‐3 and GM‐CSF as key in the network analysis, with effects most evident in females following heat exposure, warrants further investigation. Neither cytokine is typically assessed in typical cytokine panels employed in animal or human studies of heat exposure (Heled et al., 2013; Oki et al., 2022).

We also examined the pair‐fed versus control network to distinguish heat‐specific effects from those driven by food restriction. The molecular features of the pair‐fed network are consistent with established metabolic adaptations to reduced food intake, including changes in bile acid homeostasis (Chiang & Ferrell, 2019; Fu & Klaassen, 2013) and tissue‐specific alterations in inflammatory signaling (Antunes et al., 2018; Procaccini et al., 2023). In males, β‐muricholic acid was negatively correlated with serum IL‐17α, lung IL‐1α, and serum RANTES and positively correlated with serum IL‐4. In females, serum IL‐4 and serum IL‐1α were key network hubs. IL‐4 was negatively correlated with metabolism and inflammation‐related DEGs. These DEGs included, for example, *Klf15* (Krüppel‐like factor 15), a fasting‐responsive transcription factor (Gray et al., 2007; Takeuchi et al., 2024), and *Fkbp5* (FK506 binding protein 5), a glucocorticoid receptor co‐chaperone whose expression is increased in some tissues due to increased glucocorticoid levels following fasting (Fries et al., 2017; Häusl et al., 2021). Moreover, serum IL‐1α was positively correlated with UDP‐GlcNAc, a nutrient‐sensitive metabolite generated through the hexosamine biosynthetic pathway (Paneque et al., 2023), in females. These networks differ from those in heat‐exposed animals, suggesting that the heat‐induced effects discussed above are likely not due to reduced food intake.

### 4.7 Study limitations

Several limitations should be considered when interpreting these findings. Because of constraints in the incubator system, we could not collect key physiological measures, including core body temperature and behavioral changes, during heat exposure. Although rectal temperature is considered the gold standard for measuring body temperature during heat stress, we could not measure it in this study without opening the incubator and disrupting the controlled thermal environment. Because the internal thermal dose experienced by the mice is unknown, our results characterize the systemic and molecular response to a defined ambient temperature profile, not a quantified physiological heat strain. Additionally, our analysis was limited to acute endpoints assessed at the peak of the heat ramp, without evaluation of post‐exposure recovery or longer‐term effects.

## 5 CONCLUSIONS

This study investigated the systemic and pulmonary effects of a gradual ambient temperature ramp, designed to approximate the gradual temperature rise during an environmentally extreme heat event, to elucidate how this ambient temperature increase influences systemic and molecular parameters in mice. The gradual heat exposure activated a more selective pulmonary inflammatory program marked by increased chemokine and cytokine levels, rather than inducing the broad cytokine storm typical of acute heat stress. Transcriptomic profiling revealed enrichment of two immune‐related biological processes, “Immune System Process” and “Lymphocyte Activation,” in the lungs of both heat‐exposed females and males compared to controls. Exploratory multi‐omics integration identified the upregulated genes *Plac8* and *Ltb*, along with IL‐3 and GM‐CSF, as candidates for future mechanistic studies of heat‐exposure effects on the lung. Unlike earlier acute heat exposure studies, gradual heat exposure had only small effects on the cecal microbiome and the serum metabolome. Overall, our findings suggest that complex immunometabolic processes shape the pulmonary response of mice to the gradual heat‐exposure paradigm examined in this study. Because we could not quantify the physiological heat strain, our results cannot be translated into the physiological consequences of human exposure to a heatwave. Future research should clarify the temporal sequence of molecular events driving pulmonary effects after this heat‐exposure paradigm and whether these events differ by sex. Additional studies are needed to determine whether the observed immunometabolic and pulmonary responses persist, resolve, or change with varying durations and patterns of heat exposure.

## AUTHOR CONTRIBUTIONS

**Laura E. Dean:** Conceptualization, validation, formal analysis, data curation, writing—original draft, visualization. **Andrea Adamcakova‐Dodd:** Conceptualization, investigation, resources, writing—review and editing. **Hans‐Joachim Lehmler:** Conceptualization, methodology, validation, writing—review and editing, supervision, project administration, funding acquisition.

## FUNDING INFORMATION

This work was supported by grants P30 ES005605 from the National Institute of Environmental Health Science and T32 HL166134 from the National Heart, Lung, and Blood Institute. The authors thank Nicole Breese, Dr. Amanda J. Bullert, Darby Forsyth, and Dr. Hui Wang (University of Iowa) for assistance with animal exposures and tissue and sample collection. We acknowledge the support provided by the University of Iowa FOEDRC Metabolic Phenotyping Core Facility, the University of Iowa Microbiome Core for providing 16S rRNA sequencing services, and the Northwest Metabolomics Research Center (NM‐MRC) for providing metabolomics services.

## CONFLICT OF INTEREST STATEMENT

The authors have no competing interests to declare that are relevant to the content of this article.

## ETHICS STATEMENT

All experimental procedures involving animals were reviewed and approved in accordance with all Public Health Service policies and the Guide for the Care and Use of Laboratory Animals, NIH Publication No. 85–23, revised 2011, by the University of Iowa IACUC (Institutional Animal Care and Use Committee) and conform with the National Research Council's Guide for the Care and Use of Laboratory Animals (National Research Council, 1981). Details regarding the animal study are reported in accordance with the ARRIVE guidelines.

## DECLARATION OF GENERATIVE AI AND AI‐ASSISTED TECHNOLOGIES IN THE WRITING PROCESS

During the preparation of this work (July 2024–August 2026), the author(s) used Claude Opus 4.8 (Anthropic), ChatGPT (OpenAI; version 5.6), and Copilot (Microsoft; M365) to edit sections of the paper. These AI tools were used for literature searches and throughout the manuscript for editing and to help with argument flow. After using these tools/services, the authors reviewed and edited the content as needed and took full responsibility for the published article. These technologies do not hold any copyright on the work.

## Supporting information


**Table S1.** Concentrations of cytokines and chemokines (pg/mL); mean ± standard deviation.
**Table S2.** Body (g), feed (g), water (g), and relative organ weights (organ/body weight %); mean ± standard deviation.
**Figure S1.** (a) Body weight and (b) feed consumption were not significantly affected by gradual exposure to extreme heat. In contrast, (c) water consumption significantly increased in both male and female mice subjected to heat stress. Data are presented as mean ± standard deviation, and statistical significance was assessed using two‐way ANOVA with Tukey multiple comparisons.
**Figure S2.** Alpha Diversity was not altered in (a) female or (b) male mouse microbiomes after heat exposure and pair‐feeding. Beta Diversity was not changed in (c) female or (d) male mouse microbiomes after heat exposure and pair‐feeding. Alpha diversity was measured by Shannon Diversity at the feature level. PCoA and Jensen‐Shannon Divergence at the feature level were used to measure beta diversity. Alpha diversity indices and principal coordinate analysis (PCoA) plots are shown to illustrate patterns in microbial diversity and community composition among groups. No formal statistical testing was conducted for these analyses.
**Figure S3.** PCA plots showed no separation in transcriptomic profiles in the hippocampus (a) of female or (b) male mice, or in the lung (c) of female or (d) male mice, after heat exposure or pair‐feeding.
**Figure S4.** Expression patterns of differentially expressed genes (DEGs) shared across altered biological processes between female and male heat‐exposed mice and their control counterparts are shown in panels (a–d). DEG normalized counts are presented as mean ± standard deviation.

## Data Availability

The sequencing data have been uploaded to the Sequence Read Archive (https://www.ncbi.nlm.nih.gov/sra; last accessed June 2025) under the BioProject ID PRJNA1272025 and are available to the public. All other data reported in this manuscript are archived on Iowa Research Online and can be accessed at DOI: 10.25820/data.007638.
