# Supplementary material for: Ramping up the heat: Induction of systemic and pulmonary immune responses and metabolic adaptations in mice
Source: Physiol Rep. 2026 Nov 2;14(21):e71164. doi: 10.14814/phy2.71164 (PMC13728458; doi:10.14814/phy2.71164)
Supplement: Supplementary file 1 [file PHY2-14-e71164-s001.docx]

**Ramping up the Heat: Induction of Systemic and Pulmonary Immune Responses and Metabolic Adaptations in Mice**

Running title: Systemic and Pulmonary Responses to Gradual Heat

*Laura E. Dean, Andrea Adamcakova-Dodd, Hans-Joachim Lehmler^*^*

Department of Occupational and Environmental Health, College of Public Health, University of Iowa, Iowa City, IA 52242, USA

**Corresponding Author:**	Dr. Hans-Joachim Lehmler

Department of Occupational and Environmental Health

College of Public Health

University of Iowa
145 N. Riverside Drive

Iowa City, Iowa 52242

Phone: (319) 335-4981

Fax: (319) 384-4123

**E-Mail Addresses:** [laura-gosse@uiowa.edu](mailto:laura-gosse@uiowa.edu) (L. E. Dean), andrea-a-dodd@uiowa.edu (A. Adamcakova-Dodd), and [hans-joachim-lehmler@uiowa.edu](mailto:hans-joachim-lehmler@uiowa.edu) (H. Lehmler)

**Table of Contents**

| Microbiome analysis | S3 |
| --- | --- |
| RNA sequencing and analysis | S3 |
| Metabolomics LC-MS instrument parameters | S4 |
| Correlation analysis | S5 |
| **Table S1.** Concentrations of cytokines and chemokines (pg/mL); mean ± standard deviation | S7 |
| **Table S2.** Body (g), feed (g), water (g), and relative organ weights (organ/body weights %); mean ± standard deviation | S9 |
| **Figure S1**: (A) Body weight and (B) feed consumption were not significantly affected by gradual exposure to extreme heat. In contrast, (C) water consumption significantly increased in both male and female mice subjected to heat stress. | S10 |
| **Figure S2.** Alpha Diversity was not altered in (A) female or (B) male mouse microbiomes after heat exposure and pair-feeding. Beta Diversity was not changed in (C) female or (D) male mouse microbiomes after heat exposure and pair-feeding. Alpha diversity was measured by Shannon Diversity at the feature level. PCoA and Jensen-Shannon Divergence at the feature level were used to measure beta diversity. | S11 |
| **Figure S3.** PCA plots showed no separation in transcriptomic profiles in the hippocampus (A) of female or (B) male mice, or in the lung (C) of female or (D) male mice, after heat exposure or pair-feeding. | S12 |
| **Figure S4.** Expression patterns of differentially expressed genes (DEGs) shared across altered biological processes between female and male heat-exposed mice and their control counterparts are shown in panels (A-D). | S13 |
| References | S14 |

**Microbiome analysis**

DNA was extracted from each cecum sample using the DNeasy PowerLyzer PowerSoil Kit (Qiagen, Germantown, MD, USA) following the manufacturer’s instructions. DNA levels were quantified using a Nanodrop (Thermo Scientific) and stored at -80°C until further analysis. Sequencing was performed on the Illumina MiSeq platform (Illumina, San Diego, CA, USA) by amplifying the V3-V4 region of the 16S rRNA genes. Downstream analyses of fastq files were conducted using the dada2 script in R (Callahan et al., 2016; Shahi et al., 2020) to generate amplicon sequence variants (ASVs). The ASV table was analyzed using MicrobiomeAnalyst (Chong et al., 2020; Dhariwal et al., 2017). Features were filtered based on the inter-quantile range, with a minimum count of 200, a prevalence in the sample equal to 20%, and a removal percentage of 10%. No rarefication or scaling was applied to normalize the data. However, the data was transformed using relative log expression before subsequent analyses. Alpha diversity, a common measurement of microbial diversity within an experimental group, was assessed at the feature level using Shannon diversity (Kers & Saccenti, 2022). Beta diversity, a measure of microbial diversity between experimental groups, was measured using Principal Coordinates Analysis (PCoA) and Jensen-Shannon Divergence (Chen et al., 2021; Kers & Saccenti, 2022). Additionally, random forest was employed to identify differentially abundant taxa (5000 trees and 7 predictors, with randomness enabled).

**RNA sequencing and analysis**

Total RNA was isolated using the RNeasy Mini Kit (Qiagen) from the left lung lobe following the manufacturer’s instructions. RNA was checked for purity with a nanodrop (Thermo Fisher Scientific, Fair Lawn, NJ, USA). A total of 158 samples with RNA integrity values of ≥ 8.0 were submitted to Novogene (Sacramento, CA, USA) for RNA sequencing using the NovaSeq 6000 PE150 platform. Novogene conducted an additional quality control step using an Agilent 5400 Fragment Analyzer System (Agilent Technologies, Santa Clara, CA, USA), followed by cDNA library preparation employing ribosomal depletion techniques. Sequencing was then carried out to a depth of 20 million reads, with 120 base-pair paired-end sequences following the Illumina 1.9 encoding protocols established by Novogene.

Raw RNA sequencing FASTQ files were generated by Novogene through demultiplexing and concatenation for each sample. Further processing for differential gene expression was done based on methods previously described (Bullert et al., 2024). Briefly, gene counts were determined by *GenomicAlignments* (R and Rstudio version 4.2.2) using Novogene’s reference genome. Differential expression analysis was then performed using DESeq2 (version 1.38.3) (Love et al., 2014) and visualized through principal component analysis (PCA) plots using the first two principal components and volcano plots using *EnhancedVolcano* (Blighe et al., 2024). Because >30 differentially expressed genes (DEGs) were observed in heat-exposed mice, further analysis of pathways was conducted for further interpretation of the data via iPathwayGuide (Advaita Corporation, Ann Arbor, Michigan, USA) as described previously (Bullert et al., 2024).

**Metabolomics LC-MS instrument parameters**

Serum metabolomics analyses were performed at the Northwest Metabolomics Research Center (University of Washington, Seattle, WA, USA) with an AB Sciex 6500+ Triple Quadrupole MS (Sciex, Framingham, MA, USA) with an ESI ionization source and equipped with two Shimadzu UPLC pumps (Shimadzu Corporation, Kyoto, Japan) and a CTC Analytics PAL HTC-xt temperature-controlled auto-sampler (CTC Analytics AG, Zwingen, Switzerland) (Meador et al., 2020). Each sample was injected into two identical hydrophilic interaction liquid chromatography (HILIC) columns (Waters XBridge BEH Amide XP, Waters Corporation, Milford, MA, USA) on the liquid chromatography tandem mass spectrometry (LC-MS/MS) system. One column operated in ESI+ mode, and the other in ESI- mode, with mass spectrometry data acquired in the multiple-reaction monitoring (MRM) mode. The LC-MS/MS system was operated using AB Sciex Analyst 1.6.3 software, while the chromatographic peaks were integrated with AB Sciex MultiQuant 3.0.3 software. The analytical method targeted 361 metabolites and 32 stable isotope-labeled internal standards (SILISs). In this study, 185 metabolites and 30 SILISs were detected. We employed two quality control (QC) sample types: pooled human serum samples to monitor system performance and pooled study samples to assess data consistency. These QC samples were analyzed after every 10 experimental samples and demonstrated consistency with an average coefficient of variation (CV) of 2.2%.

Metabolomics data were analyzed with the MetaboAnalyst 6.0 online platform (Pang et al., 2021). Briefly, all data were normalized by sum and log-transformed. Differences between experimental groups were analyzed using Partial Least Squares Discriminant Analysis (PLS-DA), random forest modeling, and Kyoto Encyclopedia of Genes and Genomes (KEGG) pathway analysis.

**Correlation analysis**

Integrated network analyses were performed for male and female mice using xMWAS (v. 1.0) (Uppal et al., 2018). The goal of these analyses was to explore the relationships between lung gene expression, serum metabolites, serum cytokines, and lung cytokines across exposure groups (heat-exposed vs. control, heat-exposed vs. pair-fed, and pair-fed vs. control). xMWAS calculates pairwise association scores, which approximate correlation coefficients, between each cytokine level, metabolite level, or gene expression. This was accomplished using partial least squares (PLS) regression analysis. xMWAS then generated a multidata integrative network (Cao et al., 2018). Only associations with specified correlation threshold (R) and p-value (determined by Student’s t-test) were included in this network (R > 0.9 and p-value < 0.05). Finally, a multilevel community detection algorithm was applied (Blondel et al., 2008) to identify clusters genes, metabolites, and cytokines that exhibited strong internal connections but sparse external connections. Cytoscape (v. 3.10.1) was used to visualize the correlation networks from the xMWAS analyses (Otasek et al., 2019; Shannon et al., 2003).

**Table S1.** Concentrations of cytokines and chemokines (pg/mL); mean ± standard deviation

| **Cytokine**  **or**  **Chemokine** | **Sex** | **Lung Homogenate** | | | **Serum** | | |
| --- | --- | --- | --- | --- | --- | --- | --- |
|  |  | **Control**  **(n=7M, 8F)** | **Exposed**  **(n=7M, 8F)** | **Pair-Fed**  **(n=6M, 6F)** | **Control**  **(n=7M, 7F)** | **Exposed**  **(n=8M, 6F)** | **Pair-Fed**  **(n=6M, 6F)** |
| **Eotaxin** | M | 1591.9±550.9 | 1764.9±245.9 | 1538.7±144.9 | 1509.7±656.5 | 1676.1±644.2 | 1141.6±316.2 |
|  | F | 1474.6±250.1 | 1391.2±480.1 | 1269.6±242.5 | 2058.7±625.7 | 2189.9±1124.0 | 2409.6±784.0 |
| **G-CSF** | M | 1.1±0.2 | 1.1±0.4 | 1.6±0.2 | 58.7±38.2 | 67.9±41.1 | 38.4±7.2 |
|  | F | 1.3±0.5 | 1.3±0.6 | 1.0±0.3 | 32.8±9.8 | 31.2±10.6 | 34.5±15.6 |
| **GM-CSF** | M | 11.5±10.1 | 7.4±9.2 | 16.7±10.4 | 3.2±1.3 | 11.2±8.9 | 6.5±4.4 |
|  | F | 16.6±11.7 | 17.8±20.2 | 9.8±11.0 | 15.1±10.6 | 9.8±8.0 | 6.4±6.7 |
| **IFN-γ** | M | 34.9±13.2 | 39.1±8.0 | 30.7±7.9 | 2.4±1.2 | 3.7±3.7 | 2.3±2.3 |
|  | F | 34.6±8.8 | 51.5±34.8 | 32.8±2.7 | 5.1±3.5 | 6.0±3.0 | 3.1±3.0 |
| **IL-1α** | M | 21.7±7.5 | 24.4±4.6 | 20.2±6.2 | 76.3±74.1 | 22.0±15.4 | 16.7±13.4 |
|  | F | 23.6±5.9 | 35.9±31.0 | 20.6±3.4 | 15.8±6.1 | 32.4±36.6 | 21.2±12.3 |
| **IL-1β** | M | 4.5±0.9 | 4.9±1.0 | 4.7±1.4 | 3.1±6.2 | 1.2±0.6 | 1.3±0.4 |
|  | F | 5.1±0.4 | 5.8±2.0 | 4.9±1.4 | 1.2±0.4 | 0.8±0.5 | 0.9±1.2 |
| **IL-2** | M | 45.8±14.5 | 50.8±10.5 | 45.4±16.4 | 1.0±0.0 | 1.1±0.1 | 0.9±0.4 |
|  | F | 49.6±9.6 | 54.5±6.7 | 45.9±4.9 | 1.0±0.2 | 1.0±0.0 | 1.0±0.2 |
| **IL-3** | M | 2.6±1.7 | 2.3±1.9 | 4.3±2.0 | 0.4±0.2 | 3.4±7.7 | 0.4±0.1 |
|  | F | 3.3±1.7 | 2.4±2.4 | 1.9±1.6 | 0.5±0.3 | 0.8±0.5 | 0.9±1.0 |
| **IL-4** | M | 1.4±1.1 | 1.7±0.9 | 1.6±0.7 | 0.4±0.2 | 0.8±0.8 | 0.5±0.0 |
|  | F | 1.4±0.7 | 2.2±1.5 | 1.2±0.8 | 0.2±0.3 | 0.5±0.6 | 0.8±0.5 |
| **IL-5** | M | 10.4±4.5 | 9.4±3.2 | 8.9±4.0 | 1.5±1.0 | 3.0±2.4 | 2.9±1.5 |
|  | F | 8.6±3.4 | 9.8±2.2 | 10.1±2.7 | 2.6±1.9 | 2.7±1.3 | 2.4±2.0 |
| **IL-6** | M | 2.6±2.0 | 3.0±1.1 | 2.9±1.1 | 2.5±0.0 | 2.5±0.0 | 2.5±0.0 |
|  | F | 2.3±2.4 | 5.4±4.9 | 1.8±1.2 | 2.5±0.0 | 3.8±3.0 | 2.5±0.0 |
| **IL-9** | M | 44.2±21.1 | 53.6±14.9 | 34.3±16.3 | 1.7±0.6 | 2.7±2.0 | 3.8±4.5 |
|  | F | 45.5±18.6 | 75.5±61.5 | 41.1±7.7 | 3.2±2.6 | 5.3±7.9 | 3.3±2.6 |
| **IL-10** | M | 1.3±2.8 | 0.3±0.9 | 1.4±1.7 | 2.2±1.0 | 3.0±2.1 | 2.4±1.1 |
|  | F | 0.9±2.5 | 4.9±10.1 | 0.7±1.7 | 3.6±2.5 | 3.3±2.5 | 3.4±1.8 |
| **IL-12(p40)** | M | <13.7 | <13.7 | <13.7 | 261.8±116.6 | 405.4±154.6 | 232.8±73.1 |
|  | F | <13.7 | <13.7 | <13.7 | 262.6±54.1 | 374.1±75.3 | 374.1±161.3 |
| **IL-12(p70)** | M | <4.7 | <4.7 | <4.7 | 5.2±4.9 | 3.3±0.9 | 3.3±0.0 |
|  | F | <4.7 | <4.7 | <4.7 | 5.3±5.2 | 5.8±5.9 | 5.2±5.2 |
| **IL-13** | M | 16.9±16.3 | 11.9±11.7 | 7.7±15.5 | 8.4±0.5 | 7.6±1.8 | 8.5±0.0 |
|  | F | 22.8±21.8 | 39.3±42.1 | 23.0±10.4 | 7.5±2.8 | 8.1±2.5 | 7.9±1.9 |
| **IL-17α** | M | 2.1±1.2 | 1.5±0.5 | 3.2±1.7 | 17.1±11.8 | 24.4±20.2 | 16.7±9.6 |
|  | F | 2.4±1.1 | 1.9±0.7 | 1.8±1.2 | 25.9±20.9 | 20.7±11.8 | 11.0±18.0 |
| **KC** | M | 37.7±9.6 | 38.2±4.2 | 39.1±7.3 | 31.8±15.5 | 33.5±16.5 | 27.0±7.7 |
|  | F | 39.7±5.5 | 43.4±20.7 | 35.7±5.4 | 35.4±8.9 | 42.2±21.2 | 41.7±7.5 |
| **MCP-1** | M | 205.8±50.2 | 215.6±43.8 | 232.5±44.2 | 187.8±384.6 | 68.5±39.3 | 47.2±23.9 |
|  | F | 236.7±40.7 | 267.0±65.8 | 205.1±40.6 | 54.2±18.0 | 62.0±26.2 | 48.0±24.1 |
| **MIP-1α** | M | 23.9±4.1 | 33.1±8.1 | 23.3±4.5 | 0.4±0.0 | 0.5±0.2 | 0.4±0.0 |
|  | F | 30.2±9.7 | 43.9±18.2 | 27.7±10.1 | 0.4±0.1 | 0.6±0.4 | 0.4±0.2 |
| **MIP-1β** | M | 118.2±27.3 | 142.7±36.5 | 129.3±32.5 | 7.4±0.0 | 21.0±20.8 | 15.6±20.2 |
|  | F | 134.4±45.5 | 167.9±43.8 | 114.0±32.9 | 16.9±18.7 | 25.3±26.9 | 15.7±17.6 |
| **RANTES** | M | 614.1±129.8 | 833.7±204.0 | 597.3±151.5 | 21.9±14.7 | 23.2±13.7 | 7.3±5.7 |
|  | F | 922.7±684.7 | 1017.8±278.4 | 743.4±314.3 | 25.7±10.1 | 44.6±14.3 | 24.6±14.6 |
| **TNF-α** | M | 119.5±43.5 | 122.5±37.2 | 123.0±31.9 | 5.8±3.4 | 11.9±14.1 | 4.9±6.5 |
|  | F | 134.3±31.3 | 157.0±54.1 | 120.4±22.2 | 7.5±4.9 | 6.2±4.4 | 5.0±5.2 |

**Table S2.** Body (g), feed (g), water (g), and relative organ weights (organ/body weights %); mean ± standard deviation

| **Endpoint** | **Sex** | **Control**  **(n=9M, 8F)** | **Exposed**  **(n=9M, 8F)** | **Pair-Fed**  **(n=6M, 6F)** |
| --- | --- | --- | --- | --- |
| **Starting Weight** | M | 25.0±0.7 | 25.2±1.6 | 26.5±0.8 |
|  | F | 20.3±1.3 | 20.7±1.1 | 20.2±1.8 |
| **Ending Weight** | M | 24.8±0.6 | 25.3±1.8 | 26.1±0.9 |
|  | F | 20.1±1.5 | 20.4±1.2 | 19.8±1.6 |
| **Feed Consumption** | M | 0.7±0.3 | 0.8±0.3 | 0.6±0.3 |
|  | F | 0.8±0.3 | 0.8±0.3 | 0.5±0.2 |
| **Water Consumption** | M | 0.8±0.3 | 4.8±2.4 | 1.9±1.9 |
|  | F | 0.9±0.4 | 3.7±0.5 | 2.0±1.2 |
| **Brain** | M | 1.8±0.1 | 1.7±0.1 | 1.7±0.1 |
|  | F | 2.2±0.1 | 2.2±0.1 | 2.2±0.1 |
| **Lung** | M | 0.7±0.1 | 0.7±0.1 | 0.7±0.1 |
|  | F | 0.8±0.2 | 0.8±0.3 | 0.8±0.2 |
| **Liver** | M | 4.3±0.2 | 3.9±0.1 | 3.9±0.3 |
|  | F | 4.8±0.6 | 4.5±0.7 | 4.2±0.5 |
| **Combined Kidneys** | M | 1.3±0.1 | 1.3±0.1 | 1.3±0.1 |
|  | F | 1.4±0.3 | 1.4±0.2 | 1.4±0.2 |
| **Thymus** | M | 0.2±0.0 | 0.1±0.0 | 0.2±0.0 |
|  | F | 0.2±0.1 | 0.2±0.1 | 0.2±0.0 |
| **Spleen** | M | 0.3±0.0 | 0.3±0.0 | 0.3±0.0 |
|  | F | 0.4±0.1 | 0.5±0.1 | 0.4±0.1 |
| **Heart** | M | 0.5±0.1 | 0.6±0.1 | 0.6±0.1 |
|  | F | 0.6±0.1 | 0.6±0.1 | 0.6±0.1 |
| **Combined Testes/Ovary** | M | 0.8±0.0 | 0.9±0.3 | 0.8±0.0 |
|  | F | 0.4±0.4 | 0.4±0.4 | 0.4±0.4 |

**Figure S1**: (A) Body weight and (B) feed consumption were not significantly affected by gradual exposure to extreme heat. In contrast, (C) water consumption significantly increased in both male and female mice subjected to heat stress. Data are presented as mean ± standard deviation, and statistical significance was assessed using two-way ANOVA with Tukey multiple comparisons.

**Figure S2.** Alpha Diversity was not altered in (A) female or (B) male mouse microbiomes after heat exposure and pair-feeding. Beta Diversity was not changed in (C) female or (D) male mouse microbiomes after heat exposure and pair-feeding. Alpha diversity was measured by Shannon Diversity at the feature level. PCoA and Jensen-Shannon Divergence at the feature level were used to measure beta diversity. Alpha diversity indices and principal coordinate analysis (PCoA) plots are shown to illustrate patterns in microbial diversity and community composition among groups. No formal statistical testing was conducted for these analyses.

**Figure S3.** PCA plots showed no separation in transcriptomic profiles in the hippocampus (A) of female or (B) male mice, or in the lung (C) of female or (D) male mice, after heat exposure or pair-feeding.

**Figure S4.** Expression patterns of differentially expressed genes (DEGs) shared across altered biological processes between female and male heat-exposed mice and their control counterparts are shown in panels (A-D). DEG normalized counts are presented as mean ± standard deviation.

**References**

Blighe, K., Rana, S., & Lewis, M. (2024). Enhancedvolcano: Publication-ready volcano plots with enhanced colouring and labeling [R package version 1.24.0]. *Bioconductor open source software for bioinformatics*. <https://doi.org/10.18129/B9.bioc.EnhancedVolcano>

Blondel, V. D., Guillaume, J.-L., Lambiotte, R., & Lefebvre, E. (2008). Fast unfolding of communities in large networks. *J Stat Mech: Theory Exp*, *2008*(10), P10008. <https://doi.org/10.1088/1742-5468/2008/10/P10008>

Bullert, A. J., Wang, H., Valenzuela, A. E., Neier, K., Wilson, R. J., Badley, J. R., LaSalle, J. M., Hu, X., Lein, P. J., & Lehmler, H.-J. (2024). Interactions of polychlorinated biphenyls and their metabolites with the brain and liver transcriptome of female mice. *ACS Chem Neurosci*, *15*(21), 3991–4009. <https://doi.org/10.1021/acschemneuro.4c00367>

Callahan, B. J., McMurdie, P. J., Rosen, M. J., Han, A. W., Johnson, A. J., & Holmes, S. P. (2016). Dada2: High-resolution sample inference from illumina amplicon data. *Nat Methods*, *13*(7), 581–583. <https://doi.org/10.1038/nmeth.3869>

Cao, Y., Green, K., Quattlebaum, S., Milam, B., Lu, L., Gao, D., He, H., Li, N., Gao, L., Hall, F., Whinery, M., Handley, E., Ma, Y., Xu, T., Jin, F., Xiao, J., Wei, M., Smith, D., Bornstein, S., . . . Lu, S. L. (2018). Methylated genomic loci encoding microrna as a biomarker panel in tissue and saliva for head and neck squamous cell carcinoma. *Clin Epigenetics*, *10*, 43. <https://doi.org/10.1186/s13148-018-0470-7>

Chen, B., He, X., Pan, B., Zou, X., & You, N. (2021). Comparison of beta diversity measures in clustering the high-dimensional microbial data. *PLoS One*, *16*(2), e0246893. <https://doi.org/10.1371/journal.pone.0246893>

Chong, J., Liu, P., Zhou, G., & Xia, J. (2020). Using microbiomeanalyst for comprehensive statistical, functional, and meta-analysis of microbiome data. *Nat Protoc*, *15*(3), 799–821. <https://doi.org/10.1038/s41596-019-0264-1>

Dhariwal, A., Chong, J., Habib, S., King, I. L., Agellon, L. B., & Xia, J. (2017). Microbiomeanalyst: A web-based tool for comprehensive statistical, visual and meta-analysis of microbiome data. *Nucleic Acids Res*, *45*(W1), W180–W188. <https://doi.org/10.1093/nar/gkx295>

Kers, J. G., & Saccenti, E. (2022). The power of microbiome studies: Some considerations on which alpha and beta metrics to use and how to report results [Methods]. *Frontiers in Microbiology*, *12*. <https://doi.org/10.3389/fmicb.2021.796025>

Langille, M. G., Zaneveld, J., Caporaso, J. G., McDonald, D., Knights, D., Reyes, J. A., Clemente, J. C., Burkepile, D. E., Vega Thurber, R. L., Knight, R., Beiko, R. G., & Huttenhower, C. (2013). Predictive functional profiling of microbial communities using 16s rrna marker gene sequences. *Nat Biotechnol*, *31*(9), 814–821. <https://doi.org/10.1038/nbt.2676>

Love, M. I., Huber, W., & Anders, S. (2014). Moderated estimation of fold change and dispersion for rna-seq data with deseq2. *Genome Biology*, *15*(12), 550. <https://doi.org/10.1186/s13059-014-0550-8>

Meador, J. P., Bettcher, L. F., Ellenberger, M. C., & Senn, T. D. (2020). Metabolomic profiling for juvenile chinook salmon exposed to contaminants of emerging concern. *Sci Total Environ*, *747*, 141097. <https://doi.org/10.1016/j.scitotenv.2020.141097>

Otasek, D., Morris, J. H., Bouças, J., Pico, A. R., & Demchak, B. (2019). Cytoscape automation: Empowering workflow-based network analysis. *Genome Biol*, *20*(1), 185–200. <https://doi.org/10.1186/s13059-019-1758-4>

Pang, Z., Chong, J., Zhou, G., de Lima Morais, D. A., Chang, L., Barrette, M., Gauthier, C., Jacques, P., Li, S., & Xia, J. (2021). Metaboanalyst 5.0: Narrowing the gap between raw spectra and functional insights. *Nucleic Acids Res*, *49*(W1), 388–396. <https://doi.org/10.1093/nar/gkab382>

Shahi, S. K., Zarei, K., Guseva, N. V., & Mangalam, A. K. (2020). Microbiota analysis using two-step pcr and next-generation 16s rrna gene sequencing. *J Vis Exp*, *1*(152), 1–21. <https://doi.org/10.3791/59980>

Shannon, P., Markiel, A., Ozier, O., Baliga, N. S., Wang, J. T., Ramage, D., Amin, N., Schwikowski, B., & Ideker, T. (2003). Cytoscape: A software environment for integrated models of biomolecular interaction networks. *Genome Res*, *13*(11), 2498–2504. <https://doi.org/10.1101/gr.1239303>

Uppal, K., Ma, C., Go, Y. M., Jones, D. P., & Wren, J. (2018). Xmwas: A data-driven integration and differential network analysis tool. *Bioinformatics*, *34*(4), 701–702. <https://doi.org/10.1093/bioinformatics/btx656>
